# Maceration, Soxhlet Extraction, and Steam Hydrodistillation of *Anethum graveolens* Seeds: Phytochemical Profiles and Cytotoxic Effects

**DOI:** 10.3390/molecules31183337

**Published:** 2026-09-20

**Authors:** Christian Goldiș, Roxana Racoviceanu, Roxana Negrea-Ghiulai, Alexandra Mioc, Alexandra Prodea, Elisabeta Atyim, Tamara Maksimovic, Alexandra T. Lukinich-Gruia, Maria-Alexandra Pricop, Codruța Șoica

**Affiliations:** 1 Faculty of Medicine, “Victor Babeș” University of Medicine and Pharmacy, 2 Eftimie Murgu Square, 300041 Timisoara, Romania; christian.goldis@student.umft.ro; 2 Faculty of Pharmacy, “Victor Babeș” University of Medicine and Pharmacy, 2 Eftimie Murgu Square, 300041 Timisoara, Romania; roxana.ghiulai@umft.ro (R.N.-G.); alexandra.mioc@umft.ro (A.M.); alexandra.ulici@umft.ro (A.P.); elisabeta.atyim@umft.ro (E.A.); tamara.maksimovic@umft.ro (T.M.); codrutasoica@umft.ro (C.Ș.); 3 Research Centre for Experimental Pharmacology and Drug Design (X-Pharm Design), “Victor Babes” University of Medicine and Pharmacy, 2 Eftimie Murgu Square, 300041 Timisoara, Romania; 4 OncoGen Centre, Clinical County Hospital “Pius Branzeu”, Blvd. Liviu Rebreanu 156, 300723 Timisoara, Romania; alexandra.gruia@hosptm.ro (A.T.L.-G.); alexandra.pricop@oncogen.ro (M.-A.P.); 5 Department of Applied Chemistry and Environmental Engineering and Inorganic Compounds, Faculty of Industrial Chemistry, Biotechnology and Environmental Engineering, Polytechnic University of Timisoara, Vasile Pârvan 6, 300223 Timisoara, Romania

**Keywords:** dill, cytotoxic, extract, essential oil, apoptosis, mitochondrial respiration, network pharmacology, molecular docking

## Abstract

Dill, *Anethum graveolens* L., is a culinary plant used in traditional medicine to treat gastrointestinal conditions; its seeds have been previously reported to exhibit antimicrobial, anti-inflammatory, hypolipidemic, anticancer and antioxidant activities. This study aimed to assess dill seed extracts and essential oil in terms of chemical composition and cytotoxic effects, combined with an exploration of the potential underlying mechanisms. Four hydroethanolic seed extracts were prepared using Soxhlet extraction or maceration and the essential oil (AGEO) was prepared by steam distillation. Extracts were physicochemically analysed via LC-MS and spectrophotometry while the AGEO composition was determined through GC-MS. Their cytotoxic effects were assessed in A375, PANC-1 and SK-OV-3 cancer cells, while HaCaT keratinocytes were used as non-cancerous control. The mechanistic investigations included immunofluorescence assay, high-resolution respirometry, network pharmacology and molecular docking. Ferulic acid and fisetin were identified as major components of the hydroethanolic extracts, while D-limonene and carvotanacetone were the main compounds in the AGEO. All tested products were revealed to induce dose-dependent cytotoxicity (with IC_50_ values ranging between 619.8 and 810.2 μg/mL), diminished OXPHOS efficiency and a mitochondrial uncoupling effect. Morphological changes consistent with apoptosis were recorded, particularly in PANC-1 and A375 cells. Network pharmacology suggested a multitarget mechanism for EO that involved STAT3 and HSP90AA1, while the molecular docking calculations indicated that D-limonene and carvotanacetone could be accommodated within the ATP-binding pocket of Hsp90α.

## 1. Introduction

*Anethum graveolens* L., is an aromatic plant belonging to the *Apiaceae* family [1]. It contains a variety of natural products, like EO, flavonoids, phenolic and fatty acids, cardiac glycosides, saponins and tannins [2,3,4]. *A. graveolens* L. EO (AGEO) is a mixture that may contain several different compounds [5], although the chemical composition varies based on the geographical origin, harvesting time, parts of the plant and extraction method used [6]. The EO is most abundant in the flowers and seeds, and its chemical components are responsible for the plant’s biological activity [2]. Some of the bioactive compounds present in the EO include apiole, α-phellandrene, germacrene D, carvone, limonene and sabinene [2,4,7,8]. As some of the substances present in plants are non-volatile (e.g., polyphenols) [9], solvent plant-derived extracts can be prepared [10]. Therefore, *A. graveolens* L. extracts contain a series of phenolic acids and flavonoids [11,12]. Overall, *A. graveolens* L. exhibits numerous effects associated with distinct mechanisms and pathways [13]. For example, polyphenolic compounds, limonene and sabinene contribute to *A. graveolens*’s antioxidant properties [7,14], apiole and carvone lead to insecticidal and repellent activity [14,15], limonene and carvone have hypocholesterolemic and antidiabetic effects [13], while polyphenols such as quercetin show cytotoxic potential [16].

According to Mungwari et al. [17], the extract method strongly impacts the composition, yield and biological properties of plant-derived extracts and products. Furthermore, factors such as extraction duration and temperature, solvent polarity, and solid-to-solvent ratio determine the type and amounts of compounds obtained. Pașayeva et al. [18] revealed that Soxhlet extraction and maceration can return distinct phytochemical profiles that therefore have different biological effects, including cytotoxic activities. Conversely, steam hydrodistillation mainly extracts volatile compounds, producing essential oils that have a different composition compared to hydroethanolic extracts rich in non-volatile phenolic compounds. Moreover, different extraction techniques used to obtain *A. graveolens* EOs influenced the chemical composition, yield, antimicrobial and antioxidant activities of studied EOs [6]. The data from the literature indicate that non-volatile *A. graveolens* constituents are comparably affected by extraction methods. Particularly, in the case of hydroxycinnamic acid extraction from leaves of *A. graveolens*, Jevtovic et al. [19] proved that the recovery of sinapic, ferulic and chlorogenic acids was strongly influenced by ethanol concentration and the applied extraction method. These findings confirm that the extraction method is a major determinant of the phytochemical and biological properties of *A. graveolens* plant-derived extracts and products, thus supporting the comparative use of different extraction procedures. Up to now, *A. graveolens* has been widely used in the food and pharmaceutical fields for various biological applications, as a whole plant, seeds, EO, or different extracts [13]. Different studies showcase *A. graveolens*’s therapeutic potential, based on its anti-inflammatory, antioxidant, antimicrobial, antidiabetic and cytotoxic properties [20]. For example, *A. graveolens* L. exhibited favourable effects on the histology and function of the thyroid tissue [21] and reduced liver fat and hepatic oxidative stress [4], showing promising activity for diabetes management [19] and protective effects in reflux esophagitis [3]. Furthermore, *A. graveolens* L. extracts exhibited cytotoxic effects in hepatocellular carcinoma through oxidative stress induction [22]. They were associated with ROS- and caspase-dependent cell death in breast cancer and cytotoxic effects in lung and cervical cancers [20]. Meanwhile, *A. graveolens* EO led to an apoptosis-driven reduction in cell viability in the hepatocellular carcinoma line [23] and cytotoxic effects in colon carcinoma [24].

As the incidence of cancer is continuously increasing [25], so are the known disadvantages of existing treatment options, such as various side effects [20], reduced selectivity [26] and development of drug resistance; thus, new alternatives are needed. In this context, plant products exhibit lower toxicity than conventional anticancer drugs [20], demonstrate improved selectivity, can target multiple cancer pathways, and, as a result, can lead to less frequent development of resistance [27]. Additionally, plant products show synergistic effects and can be co-administered with other anticancer drugs [27]. Although the use of EOs as cytotoxic agents is a relatively new area of research, the results observed so far are promising [28]. However, although the cytotoxic effects of *A. graveolens* products have been previously reported [20,22,23,24], these effects were usually described without the investigation of the underlying mechanisms of action. Furthermore, data comparing the biological effects of *A. graveolens* products with distinct chemical compositions, obtained through different extraction techniques under identical experimental conditions, are rather limited.

To address this knowledge gap, the present study aims to provide a comparative evaluation of the cytotoxic potential of *A. graveolens* extracts obtained through different extraction techniques and AGEO in the A375 (melanoma), PANC-1 (pancreatic cancer), SK-OV-3 (ovarian carcinoma) and HaCaT (human keratinocytes) cell lines. Furthermore, the possible mechanisms of action responsible for the AGEO’s cytotoxicity were investigated using an immunofluorescence assay, complemented by computational approaches.

## 2. Results

### 2.1. Extraction Yield

Table 1 presents the sample codes, extraction conditions and extraction yields. The results indicate that the extraction yield was slightly higher for samples in which 40% ethanol was used as extraction solvent. Overall, all samples showed comparable extraction yield, ranging from 8.8 to 9.4%. AGEO obtained by hydrodistillation returned a much lower extraction yield, characteristic of EOs.

### 2.2. LC-MS Polyphenolic Profile of A1–A4

LC-MS analysis of *A. graveolens* L. seed extracts via SIM in the negative ion mode revealed a profile dominated mainly by phenolic acids. Among the quantified compounds, maceration with 60% ethanol (A2) demonstrated the highest total amount of targeted polyphenolic compounds, at about 6.03 µg/mg d.e., followed by Soxhlet extraction with 40% ethanol (A3) at about 5.46 µg/mg d.e., maceration with 40% ethanol (A1), at about 4.38 µg/mg d.e., and Soxhlet extraction with 60% ethanol (A4), at about 3.70 µg/mg d.e.

A2 extract revealed high amounts of fisetin and ferulic acid at about 2.11 and 2.08 µg/mg d.e., respectively, while p-coumaric acid exhibited the highest amounts in A2 (1.21 µg/mg d.e.) compared with A1, A3 and A4. By contrast, Soxhlet extraction with 40% ethanol (A3) selectively favoured sinapic acid extraction, at about 3.27 µg/mg d.e., which represents the highest concentration detected among all targeted compounds. Soxhlet extraction with 60% ethanol (A4) favoured the extraction of chlorogenic acid (1.99 µg/mg d.e.), compared with A2 (0.63 µg/mg d.e.), A3 (0.77 µg/mg d.e.) and A1 (below the limit of quantification).

LC-MS combined results indicate that *A. graveolens* L. seed extracts (A1–A4) revealed low-to-moderate levels of monitored phenolic compounds, mainly hydroxycinnamic acid derivatives such as ferulic, sinapic, p-coumaric and chlorogenic acids. Maceration with 60% ethanol appeared to be the most efficient general extraction method for the monitored polyphenols, whereas Soxhlet extraction exerted a more selective effect, enhancing sinapic acid recovery in 40% ethanol and chlorogenic acid recovery in 60% ethanol (Table 2). The chemical structures of the compounds detected in the A1–A4 extracts and the chromatograms obtained from LC-MS analysis are provided in the Supplementary File (Table S1 and Figure S1, respectively).

### 2.3. Total Phenolic Content, Flavonoid and Condensed Tannin Contents for A1–A4

The total phenolic content (TPC) in the extracts of *A. graveolens* was quantified using the Folin–Ciocalteu assay, expressed as mg gallic acid equivalents per gram of dry extract (mg GAE/g extract). Preliminary screening was performed for concentrations ranging between 25–500 µg/m; in the assay, 200 µg/mL extract was used to ensure absorbance values within the linear range of the calibration curve. The calibration curve was realized for gallic acid and presented linearity over the concentration range of 0–200 µg/mL, with the regression equation y = 0.0101x + 0.0059 and R^2^ = 0.9990. The test was performed in triplicate, and the results were reported as mean values ± standard deviation (Table 3).

The TPC values for the investigated samples ranged from 169.4 ± 0.5 to 177.3 ± 0.5 mg GAE/g extract. The highest value was observed in A4, followed by A2, A3 and A1. The extracts prepared with 60% ethanol, A2 and A4 showed higher values, 174.1 ± 0.3 and 177.3 ± 0.5 mg GAE/g extract, respectively. Slightly lower values were presented by the extracts prepared with 40% ethanol, A1 and A3, 169.4 ± 0.5 and 172.8 ± 0.5 mg GAE/g extract, respectively.

An aluminium chloride colorimetric assay was used to determine the total flavonoid content (TFC) of the *A. graveolens* extracts, finally expressed as mg quercetin equivalents per gram of dry extract (mg QE/g extract). Preliminary tests were performed for a concentration range of 1000–6000 µg/mL. The assay was performed for an extract concentration of 5000 µg/mL, which was selected because it provided absorbance values within the linear range of the calibration curve. The calibration curve was realized for quercetin and showed linearity over the concentration range of 0–200 µg/mL, with the regression equation y = 0.0066x + 0.0065 and R^2^ = 0.9995. The test was performed in triplicate, and the results are reported as mean values ± standard deviation (Table 4).

Among these results, A4 had the highest TFC content with 12.85 ± 0.05 mg QE/g extract, followed by A2, A3, and A1. In this case, the 60% ethanol extract, namely A4 and A2, had the highest values of TFC at 12.85 ± 0.05 and 10.05 ± 0.03 mg QE/g extract, respectively. Lower values of TFC were noticed for 40% ethanol extracts, A3 and A1, at 9.74 ± 0.03 and 5.91 ± 0.02 mg QE/g extract, respectively. Comparing the TFC and TPC, it can be observed that the values maintain a similar trend, with the highest values for both phenolic and flavonoid content for the 60% ethanol extracts.

The vanillin–HCl method was employed to establish the condensed tannin content (CTC), and the results are expressed as mg catechin equivalents per gram of dry extract (mg CE/g extract). Preliminary tests were performed for a concentration range of 2000–8000 µg/mL. The assay was performed for an extract concentration of 6000 µg/mL, which provided absorbance values within the calibration range. The calibration curve was realized for catechin, which presented very good linearity over the concentration range of 0–200 µg/mL, with the regression equation y = 0.0028x − 0.0061 and R^2^ = 0.9996. The test was performed in triplicate, and the results are reported as mean values ± standard deviation (Table 5).

The highest CTC content was observed in the case of sample A4, followed by A3, A2 and A1. The values ranged from 4.43 ± 0.03 to 9.05 ± 0.06 mg CE/g extract. Unlike the previous two assays where the 60% ethanol extracts presented the highest value, the CTC indicated that the extraction method and solvent composition could have influenced the tannin content.

### 2.4. GC-MS Composition of AGEO

The GC-MS analysis revealed a high monoterpenoid content with approximately 60.4% oxygenated monoterpenes and 39.6% monoterpene hydrocarbons. The EO profile was dominated by carvotanacetone (60.41%) and D-limonene (38.70%), both of which cumulatively accounted for 99.11% of the total chromatographic area. α-pinene, β-thujene, β-myrcene and 3-carene, all under 0.35%, can be considered minor constituents. The chemical composition is presented in Table 6; chemical structures and the chromatogram obtained from GC-MS analysis are depicted in the Supplementary File (Table S2 and Figure S2, respectively).

A cut-off of 0.10% of the total peak area was established; therefore, any peak integrating to less than 0.10% of the total chromatogram area was excluded from the final table.

### 2.5. The Effects of A. graveolens Extracts and AGEO on Cell Viability

**Figure 1:**
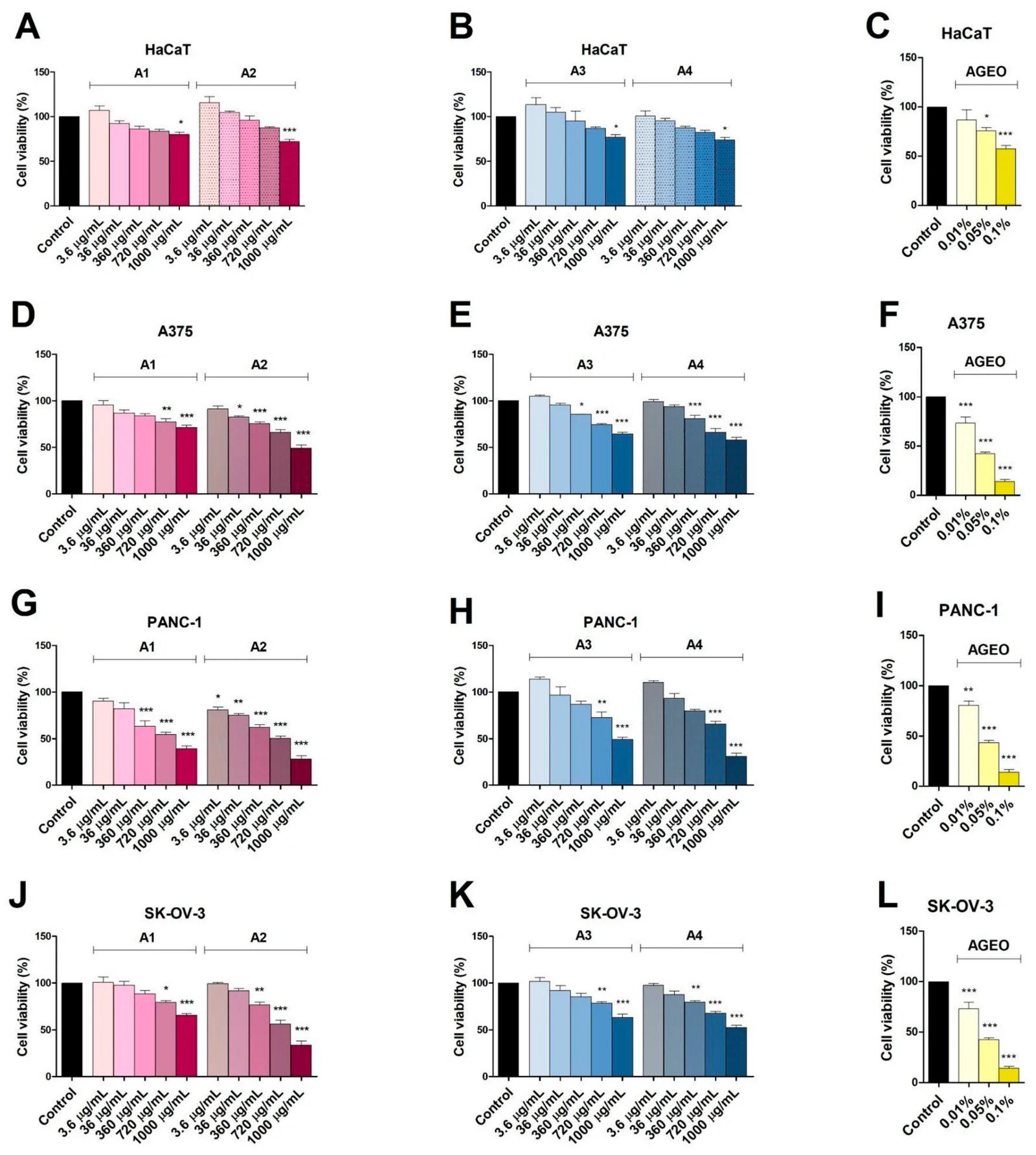
The effects of A1–4 (3.6, 36, 360, 720 and 1000 μg/mL) and AGEO (0.01, 0.05 and 0.1%) on HaCaT (**A**–**C**), A375 (**D**–**F**), PANC-1 (**G**–**I**) and SK-OV-3 (**J**–**L**) cell viability after 24 h treatment. The results are expressed as viability percentages compared to the control group, considered 100% (* *p* < 0.05; ** *p* < 0.01; *** *p* < 0.001 vs. control cells). The data represents the mean values ± SD of three independent experiments performed in triplicate.

The 24 h treatment with the highest dose of A1–4 (1000 μg/mL) induced significant decreases in HaCaT cell viability (Figure 1A,B). More precisely, the *A. graveolens* L. extracts at 1000 μg/mL decreased cell viability vs. control (100%) as follows: 80.28% ± 5.76 (A1), 72.34% ± 5.61 (A2), 77.26% ± 4.75 (A3) and 74.26% ± 4.59 (A4). In comparison, treatment with 0.05% and 0.1% AGEO reduced cells to 75.59% ± 6.84 and 57.03% ± 8.28 (Figure 1C).

In contrast to the results obtained on the HaCaT cell line, treatment with A1–4 induced a more pronounced decrease of A375 cell viability, even at lower doses. A2 had the strongest effect, decreasing cell viability in a dose-dependent manner, starting with 36 μg/mL (82.42% ± 2.34), with the strongest effect at 1000 μg/mL (49.42% ± 6.48) vs. 100% (control). This antiproliferative effect was also recorded for A3, starting at 360 μg/mL (85.94% ± 1.73) and reaching maximum at 1000 μg/mL (64.22% ± 3.39), and A4, at the same concentrations (80.92% ± 7.29 and 58.10% ± 5.77). The weakest antiproliferative effect was recorded for A1 (Figure 1D,E). In contrast, AGEO induced marked decreases of cell viability at all the concentrations tested: 0.01% (84.68% ± 2.57), 0.05% (65.10% ± 7.55) and 0.1% (33.89% ± 5.17) (Figure 1F).

On the PANC-1 cell line, all the tested extracts inhibited cell proliferation at 720 and 1000 μg/mL, the latter causing the following drop in cell viability: 39.01% ± 6.50 (A1), 28.37 ± 6.00 (A2), 49.03% ± 4.54 (A3) and 30.80% ± 6.22 (A4). Moreover, A1 was also able to significantly decrease cell viability at 360 μg/mL (63.37 ± 9.38), whereas A2 decreased cell viability at all the tested concentrations (Figure 1G,H). In the same PANC-1 cell line, treatment with 0.01%, 0.05% and 0.1% AGEO inhibited cell viability to 80.36% ± 4.25, 43.15% ± 4.03 and 14.09% ± 4.89 (Figure 1I).

In the case of the SK-OV-3 cell line, treatment with 1000 μg/mL A2 induced the strongest inhibition of cell viability (33.52% ± 7.67), followed by A4 (53.38% ± 3.71), A3 (62.98% ± 6.42) and A1 (65.56% ± 3.07). Cell viability was also decreased by lower concentrations, (360 μg/mL), following the treatment with A2 (76.69% ± 6.02) and A4 (79.85% ± 2.60). In contrast, A1 and A3 decreased cell viability only at 720 μg/mL, reaching 79.15% ± 3.73 and 78.43% ± 3.17, respectively, compared to the control group, considered to be 100% (Figure 1J,K). AGEO induced marked decreases of cell viability at all the concentrations tested: 0.01% (73.11% ± 6.12), 0.05% (42.32% ± 3.16) and 0.1% (14.02% ± 3.50) (Figure 1L).

The calculated IC_50_ values reveal that on PANC-1 cell line, A1 (763.1 μg/mL), A2 (619.8 μg/mL) and A4 (810.2 μg/mL) were the most active extracts, while on SK-OV-3 cell line, only A2 had an IC_50_ value under the tested concentration range (776.1 μg/mL)—Table 7.

To assess whether the tested extracts had a preferential cytotoxicity towards cancer cells, the selectivity index (SI) was calculated as the ratio between the IC_50_ obtained in normal HaCaT cells and the IC_50_ obtained in each cancer cell line (Table 8). The highest SI values were obtained in the PANC-1 cell line for A2 (3.138), followed by A1 (3.117), and A4 (2.613) (Table 8).

### 2.6. Immunofluorescence Evaluation of Cytoskeletal and Nuclear Alterations

**Figure 2:**
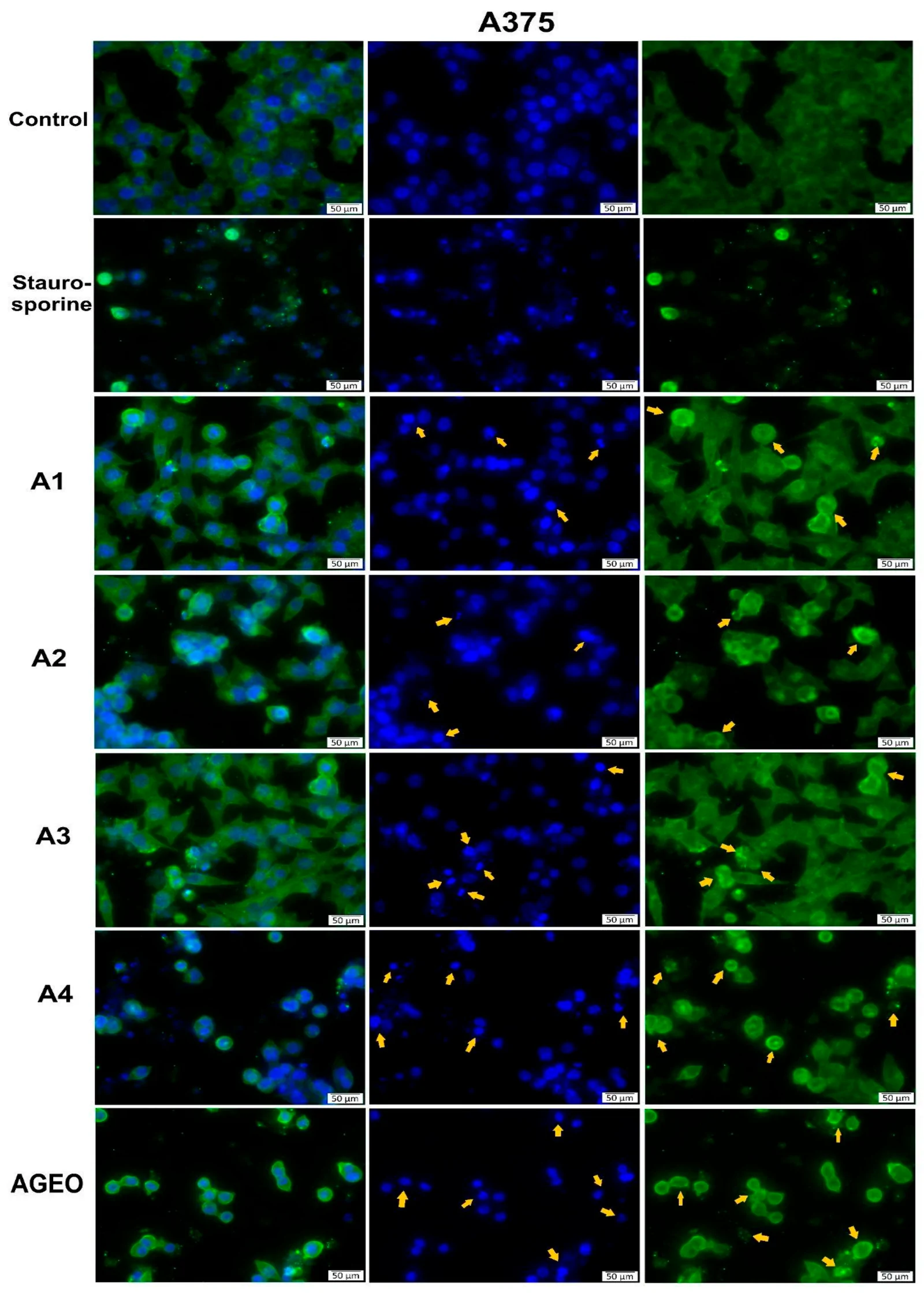
Immunofluorescence evaluation of cytoskeletal and nuclear alterations in A375 cells following 24 h treatment with 720 μg/mL A1–A4 and 0.05% AGEO. Cytoskeletal organisation was assessed by β-actin immunostaining (green), whereas nuclear morphology was evaluated following Hoechst 33342 counterstaining (blue). The morphological changes suggestive of apoptosis-like cell death are indicated by yellow arrows. Images were acquired at 40× magnification; scale bar was 50 μm.

**Figure 3:**
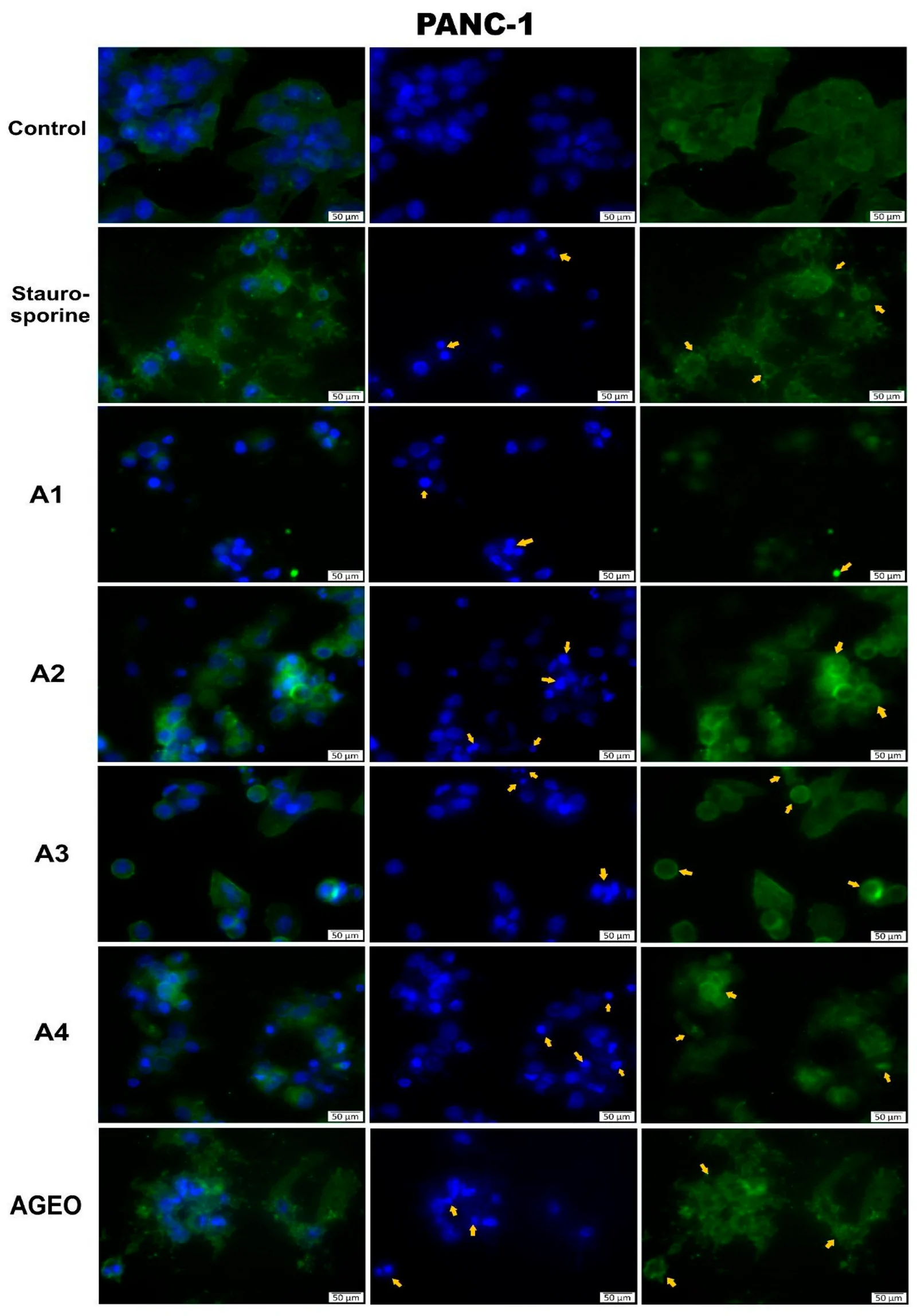
Immunofluorescence evaluation of cytoskeletal and nuclear alterations in PANC-1 cells following 24 h treatment with 720 μg/mL A1–A4 and 0.05% AGEO. Cytoskeletal organisation was assessed by β-actin immunostaining (green), whereas nuclear morphology was evaluated following Hoechst 33342 counterstaining (blue). The morphological changes that suggest apoptosis-like cell death are indicated by yellow arrows. Images were acquired at 40× magnification; the scale bar was 50 μm.

**Figure 4:**
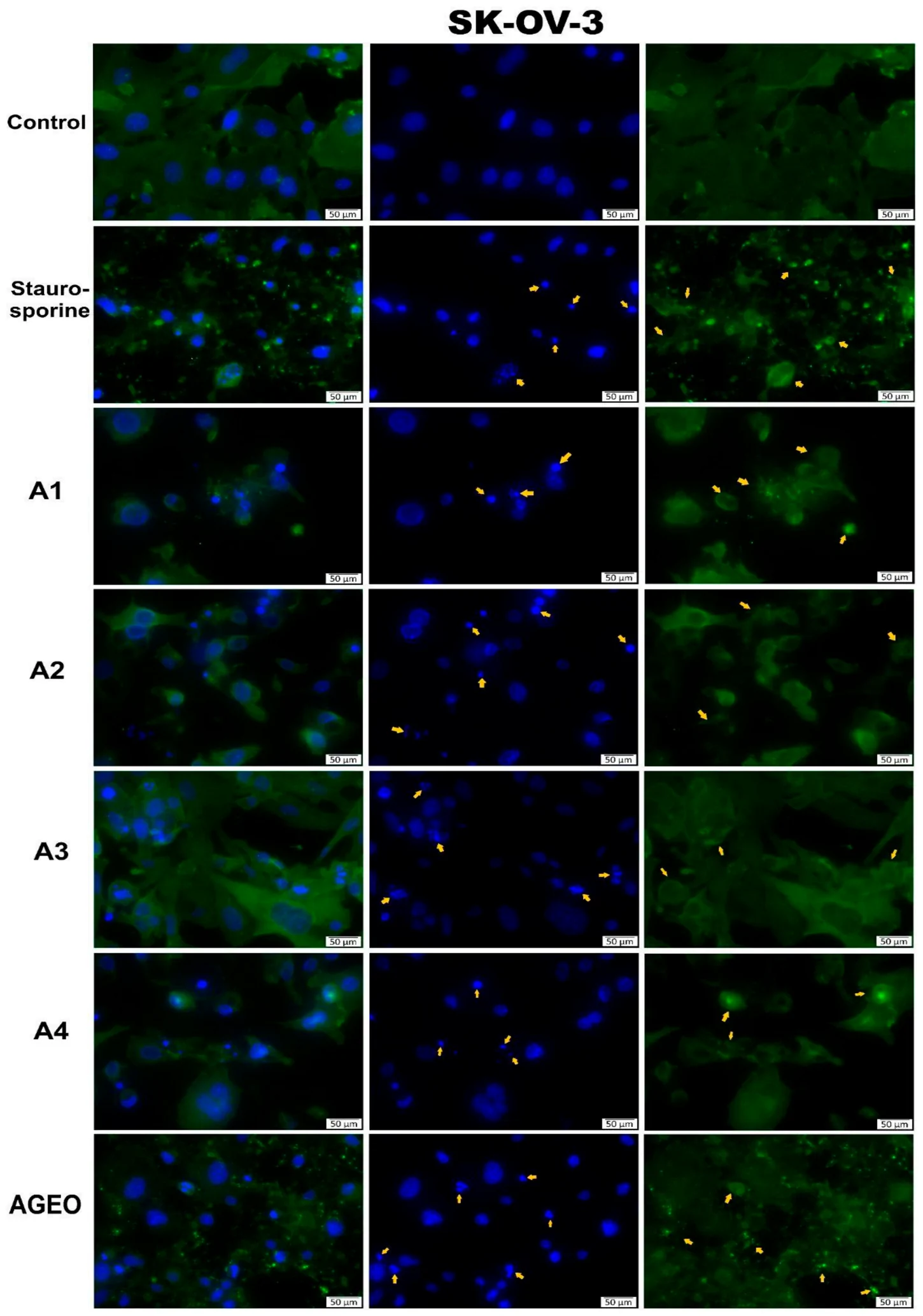
Immunofluorescence evaluation of cytoskeletal and nuclear alterations in SK-OV-3 cells following 24 h treatment with 720 μg/mL A1–A4 and 0.05% AGEO. Cytoskeletal organisation was assessed by β-actin immunostaining (green), whereas nuclear morphology was evaluated following Hoechst 33342 counterstaining (blue). The morphological changes that suggest apoptosis-like cell death are indicated by yellow arrows. Images were acquired at 40× magnification; the scale bar was 50 μm.

The immunofluorescence analysis of A375, PANC-1 and SK-OV-3 cells revealed that A1–4 induced morphological alterations compared with the untreated cells. Control cells were adherent and normally spread, with a normal cell-specific shape, a well-organised cytoskeletal network and mostly regular oval nuclei. Treatment with A1–A4 induced various degrees of cytoskeletal remodelling and nuclear damage in all cell lines. Comparison revealed that extracts A1 and A3 produced moderate changes, such as partial loss of β-actin organisation, random cell rounding and limited nuclear condensation. However, more pronounced alterations were observed after A2 and A4 treatment. These changes included reduced cell spreading, rounding and detachment of cells, diffuse/punctate β-actin staining and increased nuclear condensation or fragmentation. AGEO treatment induced similar marked morphological alterations compared with the expected untreated cells. The cytoskeletal organisation was disrupted and predominantly punctate, with reduced cell spreading, rounded morphology, condensed/fragmented nuclei and randomly small fragmented nuclear bodies; these morphological changes possibly suggest apoptosis-like cell death. The staurosporine-treated cells served as a positive control for cell death. Overall, these findings suggest that A1–A4 and AGEO induced morphological changes that could indicate apoptosis in the tested cell lines (Figure 2, Figure 3 and Figure 4).

### 2.7. Evaluation of Mitochondrial Respiration

**Figure 5:**
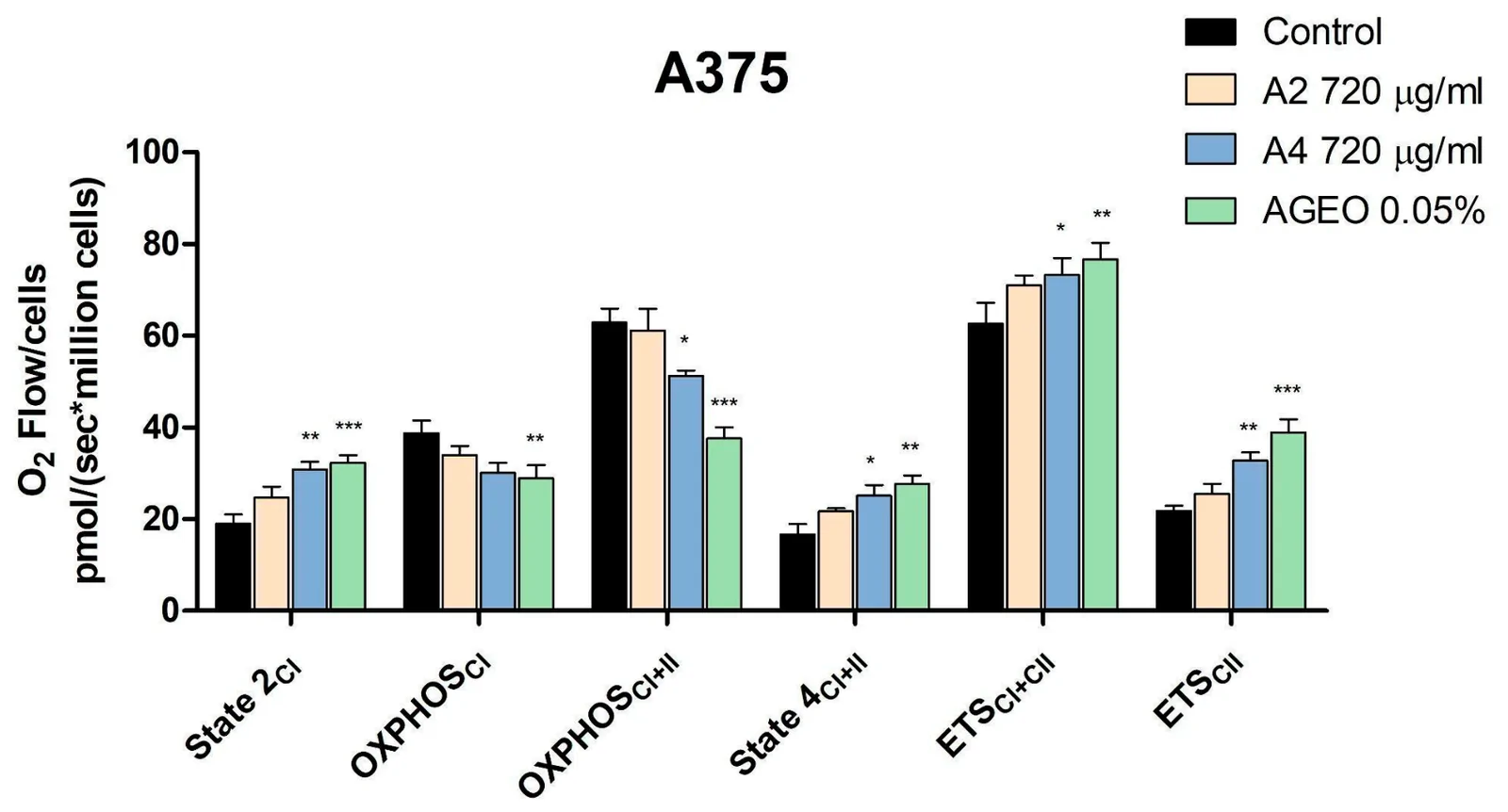
High-resolution respirometry analysis of A375 cells treated with A2, A4 (720 μg/mL) and AGEO (0.05%). Oxygen consumption was recorded during the assessment of Complex I-linked respiration (State 2CI), OXPHOS capacity supported by Complex I (OXPHOSCI) and by the conjunctive Complex I+II (OXPHOSCI+II), LEAK respiration (State 4CI+II) and the maximal electron transport system capacity after support by Complex I and II substrates (ETSCI+II) and Complex II alone (ETSCII). Results are expressed as mean values ± SD of three independent experiments (* *p* < 0.05, ** *p* < 0.01 and *** *p* < 0.001).

In A375 cells, treatment with A4 and AGEO significantly altered mitochondrial respiration compared to control. A2 treatment induced similar changes in mitochondrial respiratory rates, however, without reaching statistical significance. In particular, State 2CI respiration increased from 19.04 ± 3.46 in control cells to 30.87 ± 3.91 and 32.25 ± 4.01 after A4 and AGEO treatment. These results indicate that A4 and AGEO can enhance the non-phosphorylating Complex I-linked respiration. Conversely, OXPHOSCI significantly decreased only in the case of AGEO, from 38.81 ± 4.62 to 28.96 ± 4.9, while OXPHOSCI+II decreased from 62.37 ± 5.23 to 51.24 ± 3.04 after A4 treatment and to 37.58 ± 4.21 after AGEO treatment, suggesting impaired ADP-dependent ATP-producing respiration. State 4CI+II, also called LEAK respiration, increased from 16.77 ± 3.85 in control cells to 25.09 ± 4.03 and 27.83 ± 3.31 in cells treated with A4 and AGEO; these results support the presence of increased proton leak and reduced coupling efficiency. In parallel, maximal ETSCI+II increased from 62.64 ± 7.92 to 73.06 ± 6.31 and 76.28 ± 6.17 and ETSCII increased from 21.81 ± 2.91 to 32.62 ± 3.51 and 38.99 ± 4.82 (Figure 5). Taken together, these data indicate an uncoupling-like mitochondrial effect together with reduced OXPHOS efficiency.

**Figure 6:**
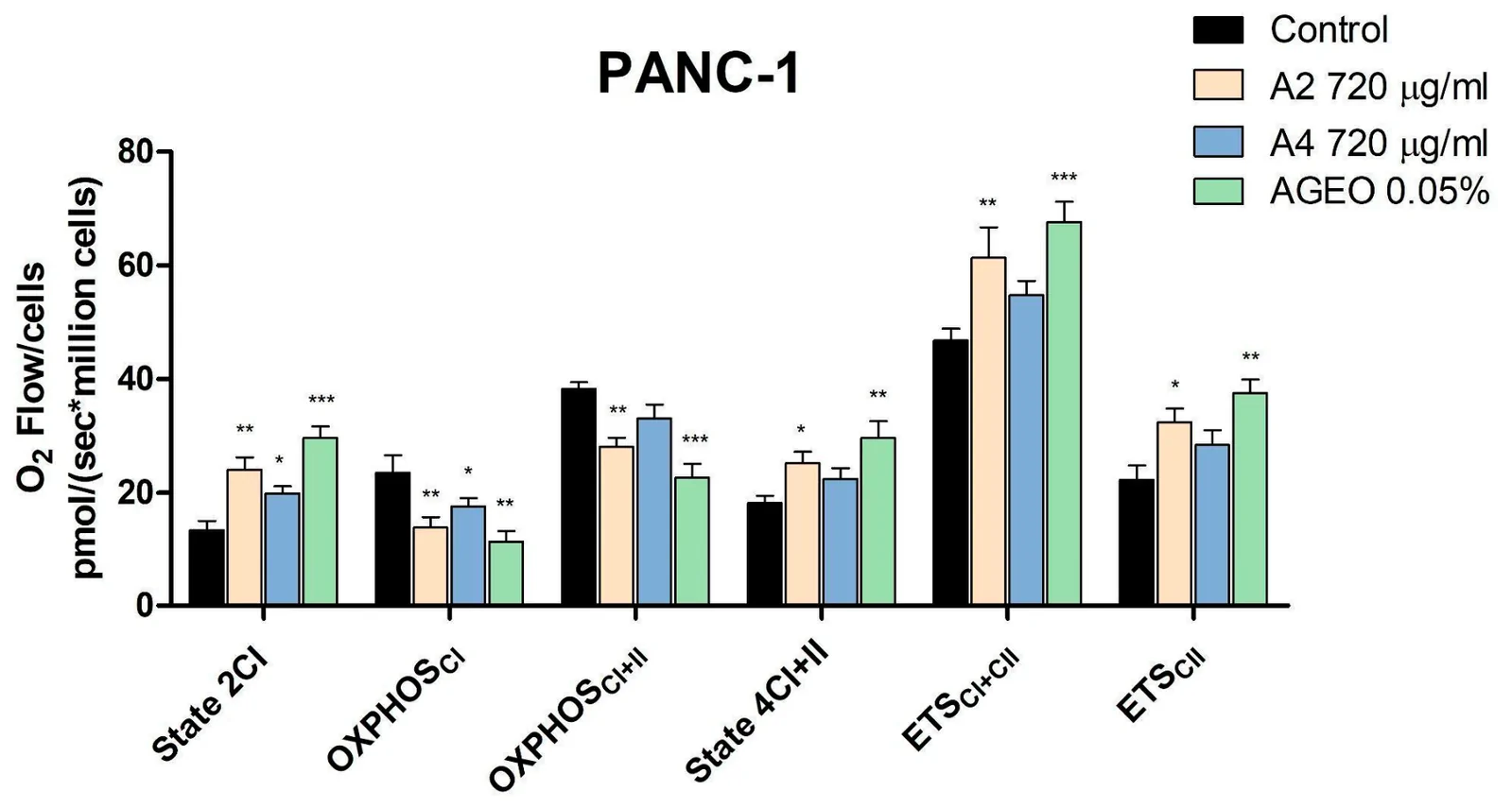
High-resolution respirometry analysis of PANC-1 cells treated with A2, A4 (720 μg/mL) and AGEO (0.05%). Oxygen consumption was recorded during the assessment of Complex I-linked respiration (State 2CI), OXPHOS capacity supported by Complex I (OXPHOSCI) and by the conjunctive Complex I+II (OXPHOSCI+II), LEAK respiration (State 4CI+II) and the maximal electron transport system capacity after support by Complex I and II substrates (ETSCI+II) and Complex II alone (ETSCII). Results are expressed as mean values ± SD of three independent experiments (* *p* < 0.05, ** *p* < 0.01 and *** *p* < 0.001).

In PANC-1 cells, treatment with A2, A4 and AGEO altered mitochondrial respiration in a similar pattern to that observed in the A375 cell line, thus suggesting the presence of the uncoupling effect together with inhibition of active respiration. More precisely, State 2CI respiration increased from the control value of 13.64 ± 2.83 to 24.09 ± 3.71, 19.73 ± 2.25 and 29.68 ± 3.51 after A2, A4 and AGEO treatment. OXPHOSCI decreased from 23.51 ± 5.30 (control) to 13.93 ± 3.15 (A2), 17.63 ± 2.52 (A4) and 11.41 ± 3.02 (AGEO) while OXPHOSCI+II significantly decreased from 38.05 ± 2.14 (control) to 28.09 ± 2.73 (A2), and 22.18 ± 4.16 (AGEO). In parallel, State 4CI+II significantly increased from 18.27 ± 2.19 (control) to 25.22 ± 4.55 (A2), and to 29.63 ± 5.04 (AGEO) indicating enhanced proton leak and reduced coupling efficiency. Maximal ETSCI+II also significantly increased from 46.82 ± 3.81 (control) to 61.82 ± 9.20 (A2), and 67.60 ± 7.09 (AGEO), while ETSCII increased from 22.41 ± 4.37 (control) to 32.31 ± 3.51, and 37.09 ± 4.61 after the treatment with A2, A4 and AGEO (Figure 6). Overall, these results suggest that the treatments, particularly AGEO, impair mitochondrial coupling efficiency rather than directly inhibiting the electron transport chain.

**Figure 7:**
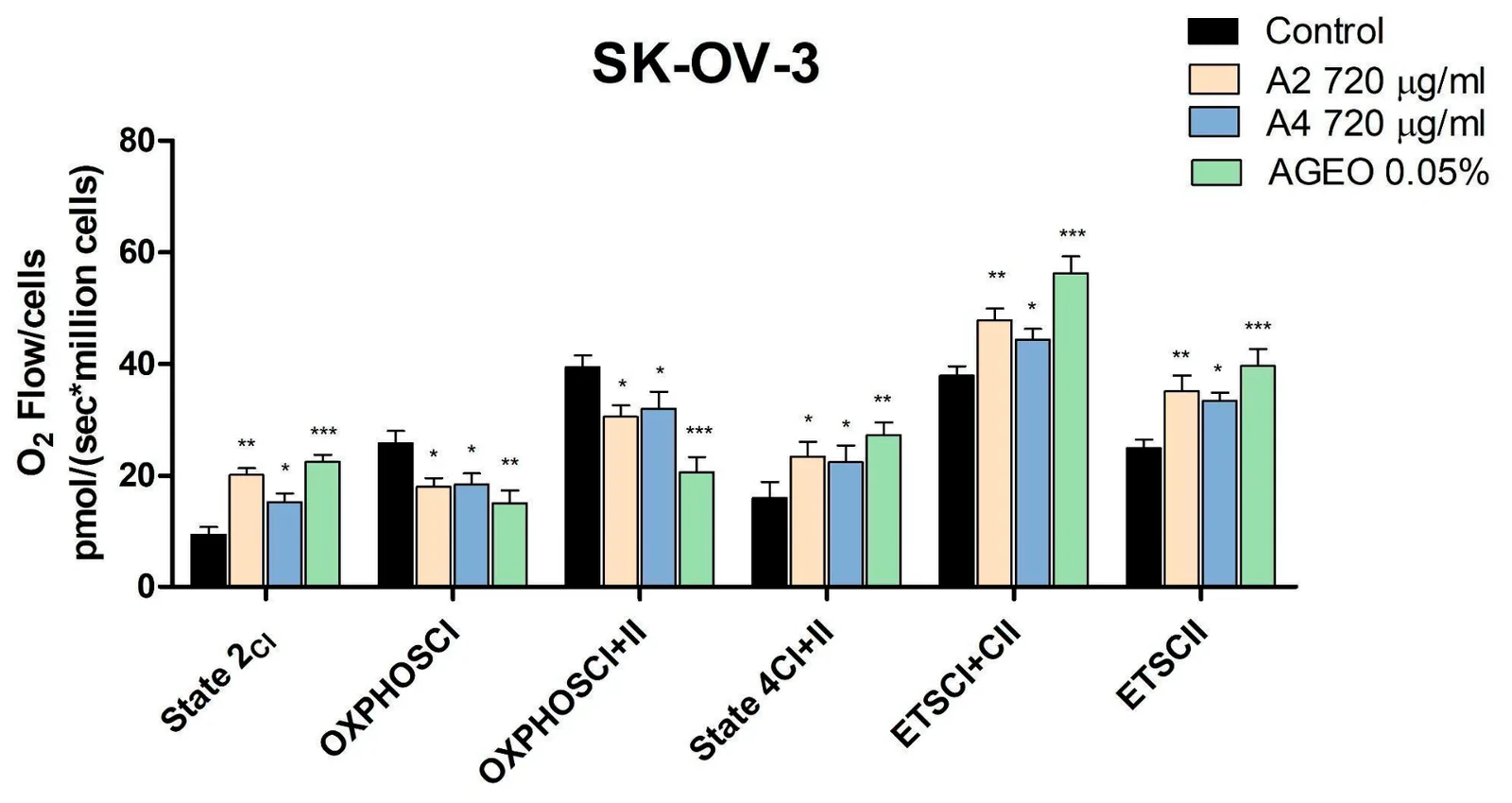
High-resolution respirometry analysis of SK-OV-3 cells treated with A2, A4 (720 μg/mL) and AGEO (0.05%). Oxygen consumption was recorded during the assessment of Complex I-linked respiration (State 2CI), OXPHOS capacity supported by Complex I (OXPHOSCI) and by the conjunctive Complex I+II (OXPHOSCI+II), LEAK respiration (State 4CI+II) and the maximal electron transport system capacity after support by Complex I and II substrates (ETSCI+II) and Complex II alone (ETSCII). Results are expressed as mean values ± SD of three independent experiments (* *p* < 0.05, ** *p* < 0.01 and *** *p* < 0.001).

Similar findings were observed for the SK-OV-3 cell line; treatment with A2, A4 and AGEO induced changes of respiratory rates suggestive of the uncoupling effect. State 2CI and State 4CII respiration increased from 9.44 ± 2.22 and 16.03 ± 4.95 in control cells to 20.13 ± 2.09 and 23.47 ± 4.60 (A2), 15.30 ± 2.59 and 22.85 ± 5.17 (A4) and 22.50 ± 2.90 and 27.27 ± 4.00 (AGEO). OXPHOSCI and OXPHOSCI+II decreased from 25.81 ± 3.80 and 39.46 ± 3.50 (control) to 18.04 ± 2.71 and 30.59 ± 3.16 (A2), 18.97 ± 3.90 and 32.02 ± 4.21 (A4) and to 15.13 ± 3.93 and 20.51 ± 4.73 (AGEO). Treatment with A2, A4 and AGEO also increased maximal ETSCI+II from 37.58 ± 4.06 (control) to 47.92 ± 4.52, 44.09 ± 4.30 and 56.28 ± 5.2 while ETSCII increased from 24.95 ± 2.73 to 35.13 ± 4.08, 34.33 ± 3.18 and to 39.71 ± 5.16 after the treatment with A2, A4 and AGEO (Figure 7). Overall, these results indicate that the treatments, particularly AGEO, reduce mitochondrial coupling efficiency while preserving or enhancing maximal electron transport capacity.

### 2.8. Evaluation of MMP

**Figure 8:**
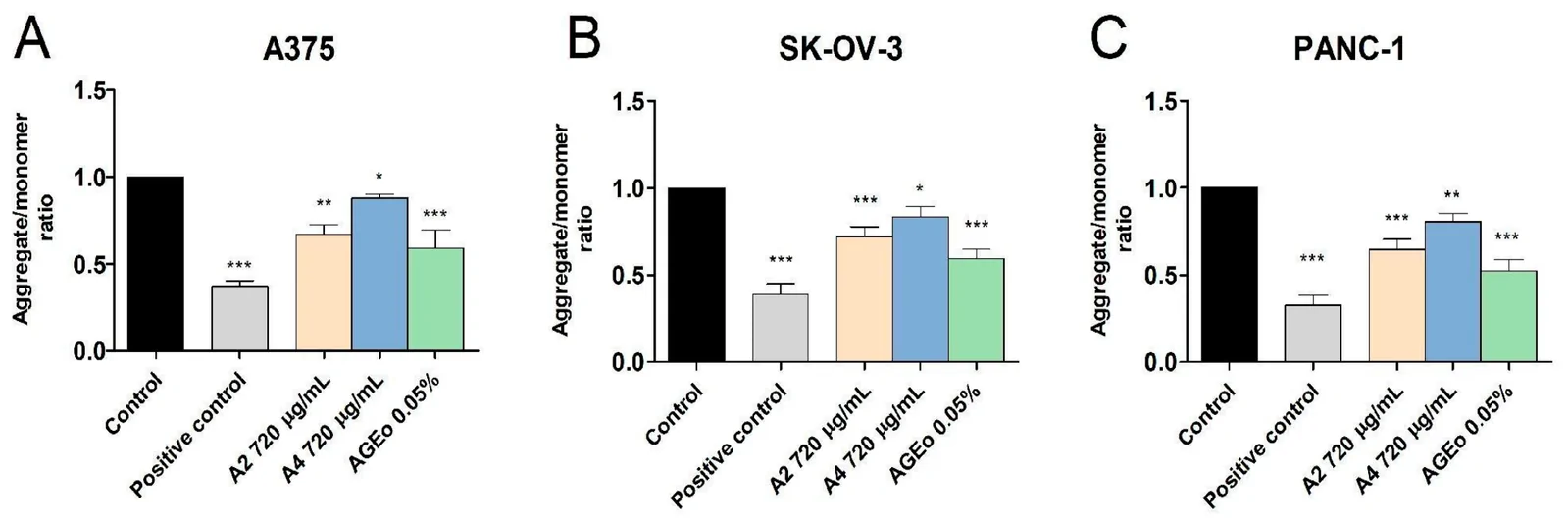
The MMP of A375 (**A**), SK-OV-3 (**B**) and PANC-1 (**C**) cells exposed for 24 h to A2, A4 (720 μg/mL) and AGEO (0.05%). FCCP (50 μM) was used as positive control. The results are expressed as mean  ±  SD of the JC-1 aggregate/monomer ratio; *n*  =  3 per group; * *p* < 0.05, ** *p* < 0.01 and *** *p* < 0.001.

The JC-1 assay was used to evaluate the effect of A2, A4 and AGEO on MMP. The results presented in Figure 8 show that both extracts (A2 and A4) as well as AGEO were able to decrease the JC-1 aggregate/monomer (red/green) ratio vs. control in all tested cancer cell lines, thus suggesting the presence of mitochondrial depolarization. Specifically, the effect was stronger in PANC-1 cells, with AGEO (0.522 ± 0.06) inducing the strongest depolarization vs. 1 (control), followed by A2 (0.642 ± 0.05) and A4 (0.807 ± 0.04)—Figure 8C. In A375 cells, treatment with A2, A4 and AGEO decreased the aggregate/monomer ratio vs. control (1) as follows: 0.670 ± 0.05, 0.874 ± 0.03 and 0.591 ± 0.10 (Figure 8A). Similar findings were observed for SK-OV-3, where treatment with A2, A4 and AGEO decreased the aggregate/monomer ratio to 0.719 ± 0.05, 0.836 ± 0.05 and 0.587 ± 0.05 (Figure 8B).

### 2.9. Target Identification and Network Pharmacology of AGEO in Pancreatic Cancer

**Figure 9:**
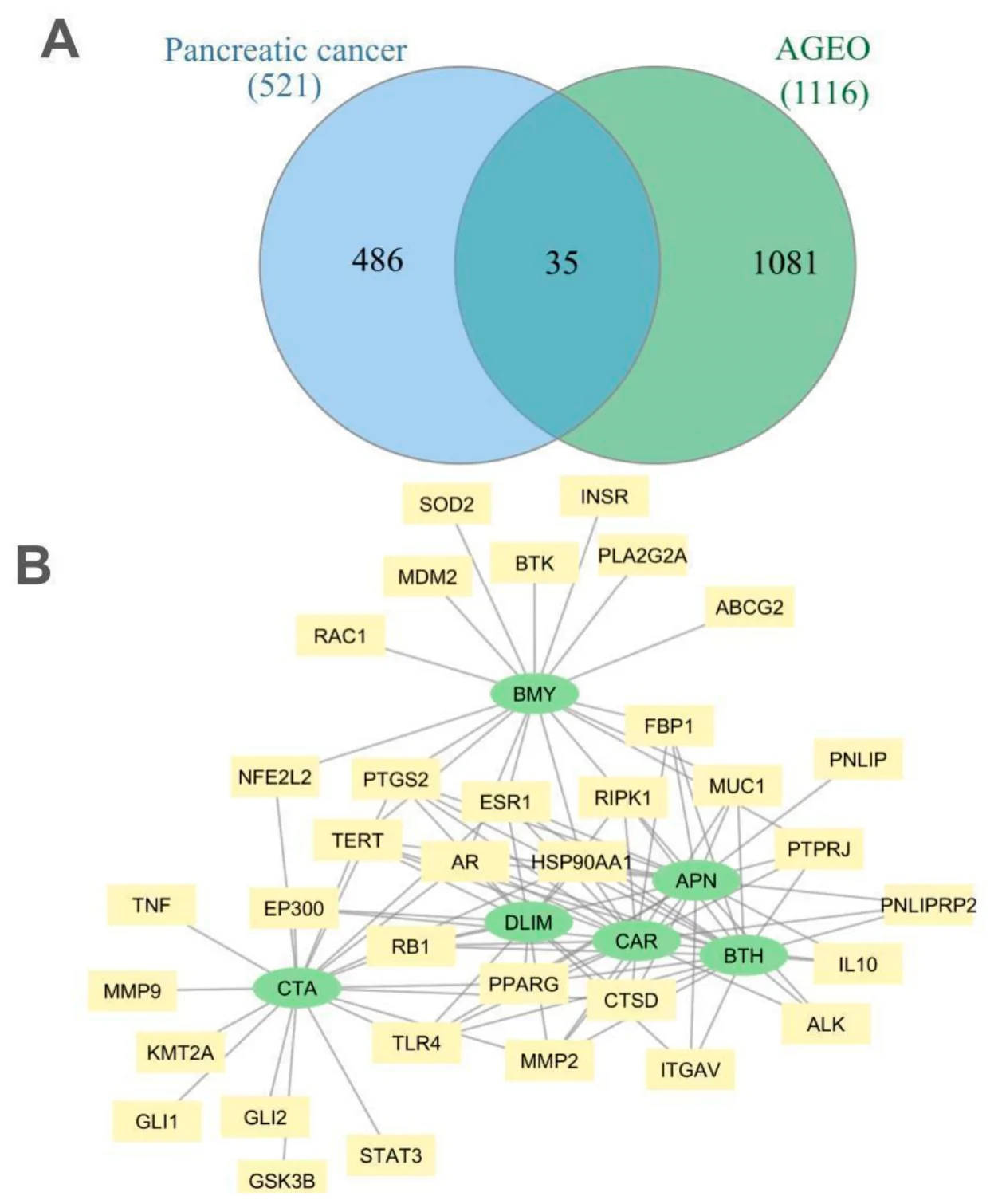
(**A**): Venn diagram illustrating the overlap between the predicted protein targets (Swiss Target Prediction and PharmMapper) of the AGEO constituents and proteins encoded by genes relevant in pancreatic cancer (GeneCards); (**B**): Compound–target network (Perfused force directed layout, 41 nodes, 104 edges) depicting the predicted interactions between AGEO constituents (APN—α-pinene, BTH—β-thujene, BMY—β-myrcene, CAR—3-carene, DLIM—D-limonene and CTA—carvotanacetone) and protein targets.

An in silico analysis was used to identify potential mechanisms of action of AGEO in pancreatic cancer, a type of cancer selected based on the increased cytotoxicity observed for AGEO in the PANC-1 cell line. The predicted targets for the AGEO constituents, α-pinene, β-thujene, β-myrcene, 3-carene, D-limonene and carvotanacetone, were intersected with proteins encoded by pancreatic cancer-relevant genes according to GeneCards, resulting in 35 predicted common targets (Figure 9A). To visualise how individual components of AGEO might interact with the predicted target, a compound–target network with 41 nodes and 104 edges was constructed in Cytoscape (Figure 9B). The network indicates that α-pinene, β-thujene, 3-carene, and D-limonene largely target the same proteins, suggesting they may exert similar pharmacological effects through shared molecular targets. Meanwhile, β-myrcene and carvotanacetone not only share several targets with these compounds but also might bind to specific targets, suggesting they may add additional biological activities through different mechanisms of action.

**Figure 10:**
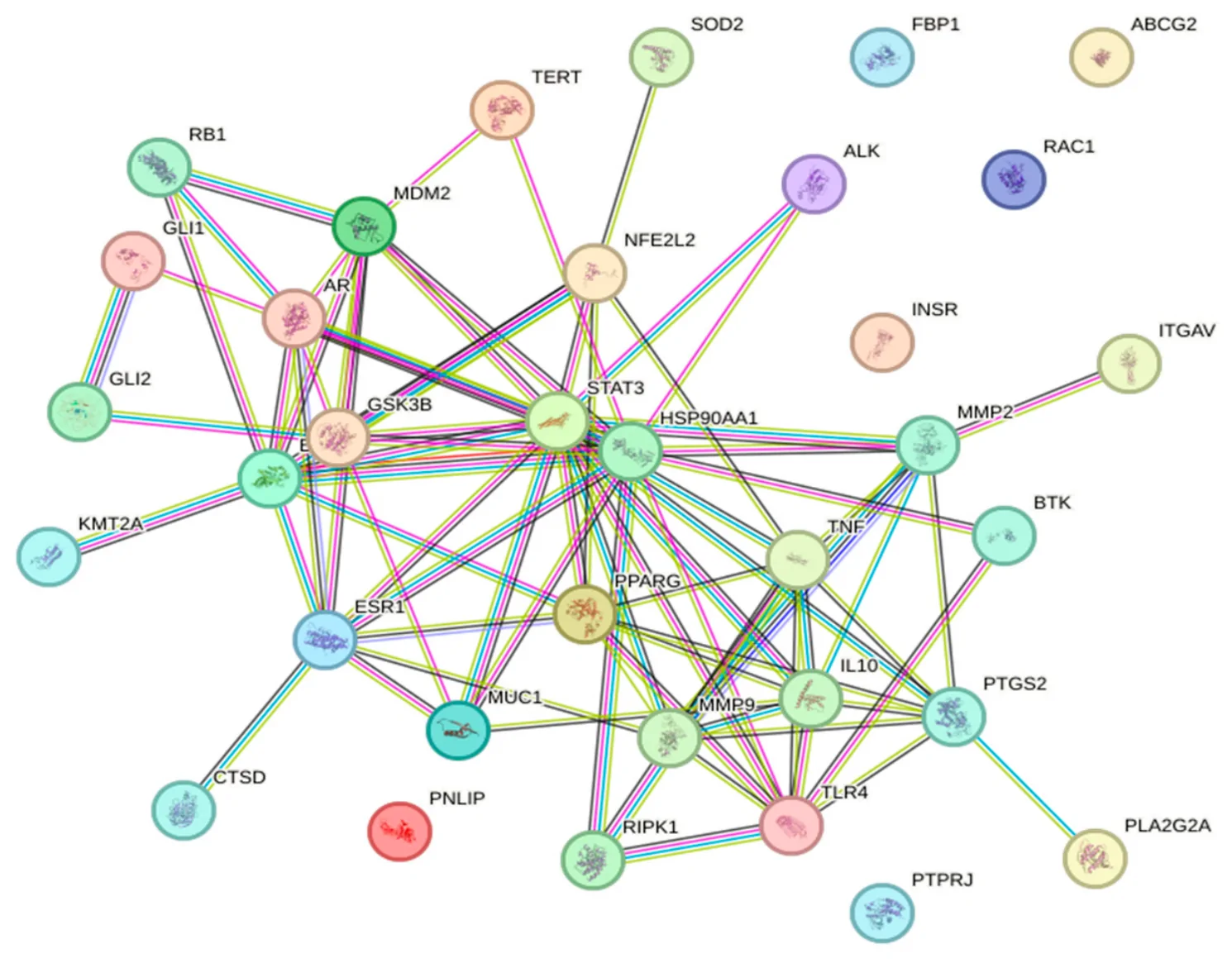
STRING protein–protein interaction network of the 35 shared targets at a high-confidence interaction threshold (>0.7) and an FDR OF 5%. Edge colors denote the evidence sources supporting protein–protein interactions in the STRING database (turquoise: curated databases; pink: experimental validation; green: gene neighborhood; purple: protein homology).

To identify biologically relevant interactions among the predicted targets of AGEO constituents, the interactions among the 35 target proteins were analysed in STRING with high confidence (>0.700) and a false discovery rate (FDR) of 5% (Figure 10). The STRING results showed that six protein targets appeared as discontinued nodes, namely, PNLIP, PTPRJ, INSR, RAC1, FBP1 and ABCG2, and these were thus eliminated from the subsequent analysis.

**Figure 11:**
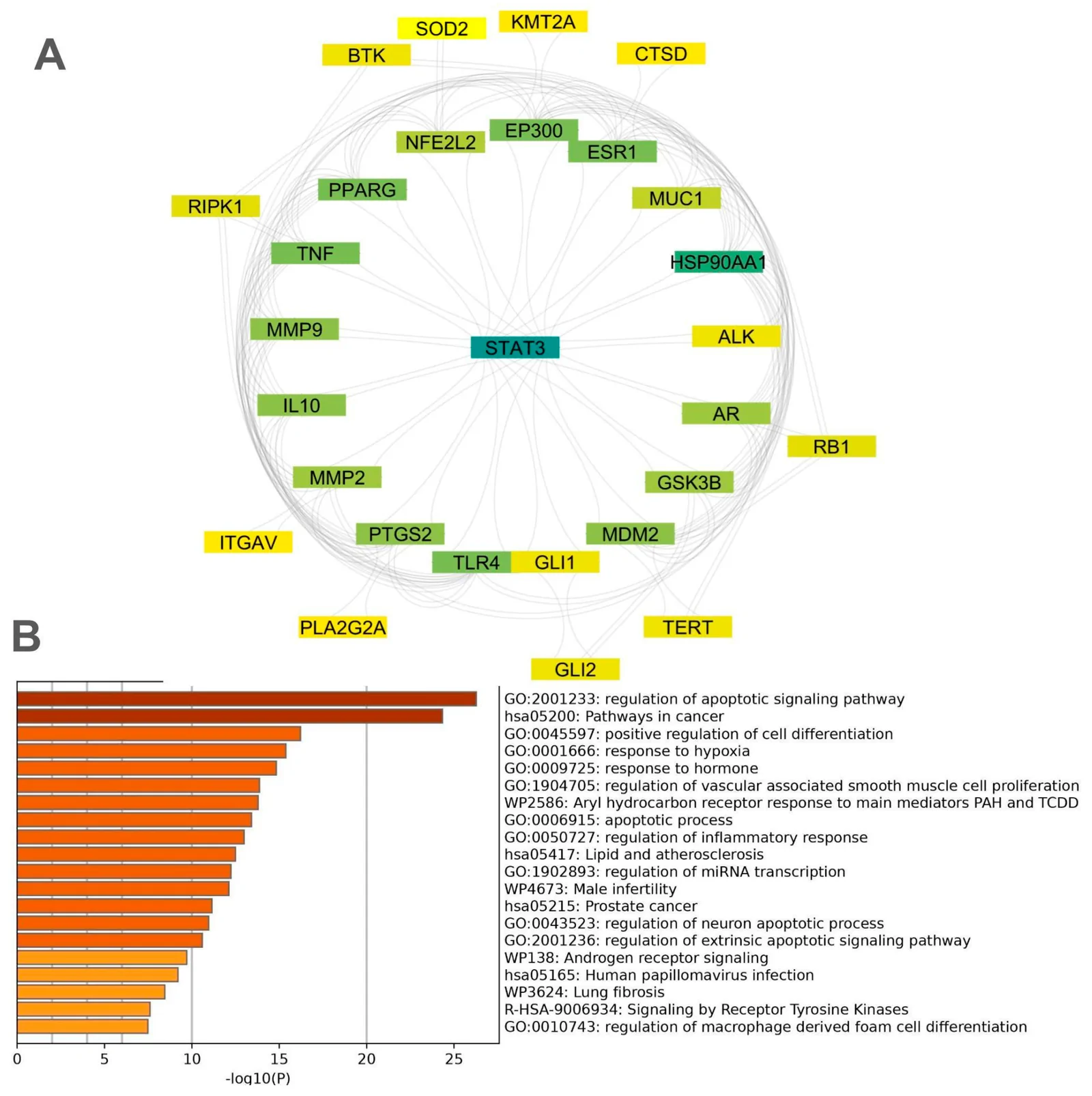
(**A**): Protein–protein interaction network using yfiles radial layout (28 nodes, 160 edges). The gradient color of the nodes, from yellow to blue, is proportional to the degree score of the node in the network; (**B**): Enrichment analysis of the 28 nodes showing the top biological pathways and processes from KEGG Pathway, GO Biological Processes, and WikiPathways databases. Enrichment significance is expressed as −log_10_(P), with longer bars indicating greater statistical significance.

The remaining 28 nodes were used to construct a protein–protein interaction network with 160 edges, a clustering coefficient of 0.475 and a network density of 0.212 (Figure 11A). Among the highly connected nodes in the network, depicted in blue and dark green boxes, were STAT3, HSP90AA1, TNF, PPARG and TLR4 (Figure 11A and Table 9), which also had superior closeness centrality (CC) and betweenness centrality (BC) scores, suggesting they might act as key regulators in the network. Furthermore, the top 10 hub targets of the network were ranked using the maximal clique centrality (MCC) algorithm in CytoHubba. Based on the network topology characteristics summarised in Table 7, STAT3 and HSP90AA1 were selected as potential primary targets for AGEO, based on their presence in the top 10 MCC-ranked list with the highest CC and BC scores. Although the network suggested the superior role of STAT3 compared to HSP90AA1 in the network, because STAT3 was predicted as a potential target exclusively for carvotanacetone, whereas HSP90AA1 was predicted as a common target for all constituents of the AGEO, heat shock protein 90α (HSP90α) encoded by *HSP90AA1* was selected for subsequent molecular docking studies.

Metascape was used to conduct an enrichment analysis of the genes encoding the 28 protein targets to discover relevant biological processes and signalling pathways connected with the predicted target proteins, thus providing insight into the potential mechanisms of AGEO constituents. Among the top 20 enriched processes and pathways, regulation of the apoptotic signalling pathway and pathways in cancer were significantly enriched, while regulation of the extrinsic apoptotic pathway was moderately enriched, suggesting that the predicted targets might regulate apoptosis (Figure 11B).

### 2.10. Molecular Docking

Based on the network pharmacology analysis, HSP90AA1 was identified as a potential target shared by all six constituents of AGEO. Therefore, Hsp90α, the protein encoded by *HSP90AA1*, was selected for molecular docking to investigate whether these constituents could be accommodated within its binding site. Molecular docking was performed using Hsp90α (PDB ID: 6LR9). The co-crystallised ligand EOR yielded a docking score of −8.6 kcal/mol and was used as the reference. Among the investigated phytochemicals, D-limonene and carvotanacetone exhibited the most favourable docking scores (−7.2 kcal/mol each), followed by 3-carene (−6.0 kcal/mol), β-thujene (−5.8 kcal/mol), β-myrcene (−5.8 kcal/mol) and α-pinene (−5.4 kcal/mol) (Table 10). The two compounds with the most favourable docking scores, D-limonene and carvotanacetone, were also the predominant constituents of the EO, accounting for approximately 38.7% and 60.4% of the total volatile composition, respectively.

**Figure 12:**
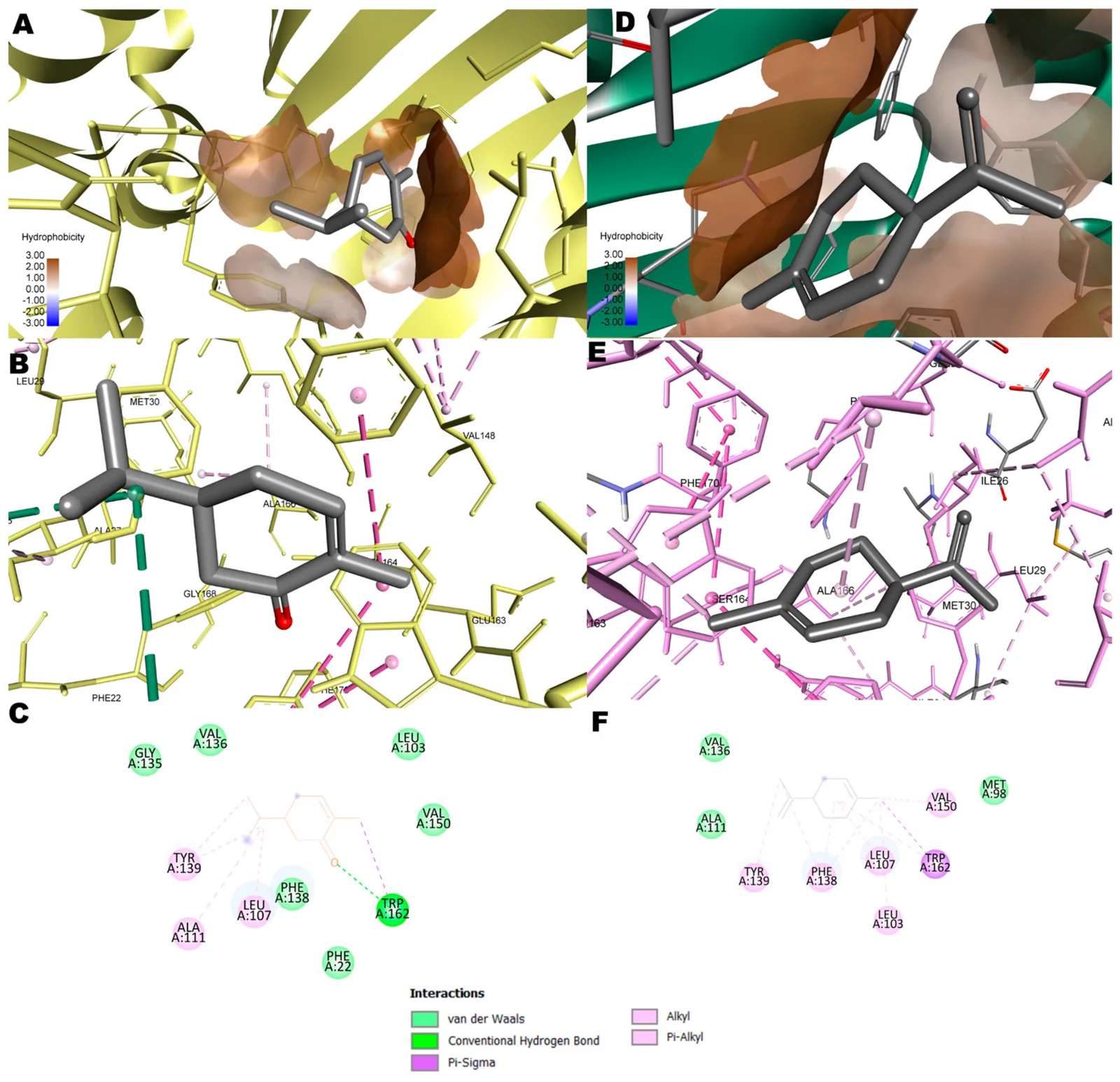
Predicted binding modes of carvotanacetone and D-limonene within the Hsp90α binding pocket. (**A**) Hydrophobicity surface of carvotanacetone. (**B**) Three-dimensional interaction profile of carvotanacetone. (**C**) Two-dimensional interaction diagram of carvotanacetone. (**D**) Hydrophobicity surface of D-limonene. (**E**) Three-dimensional interaction profile of D-limonene. (**F**) Two-dimensional interaction diagram of D-limonene.

The predicted binding mode of carvotanacetone within the Hsp90α binding pocket is illustrated in Figure 12A–C. The ligand occupies a predominantly hydrophobic region of the binding site (Figure 12A), where it is stabilised mainly through hydrophobic contacts, including alkyl, π-alkyl and π-sigma interactions with surrounding residues (Figure 12B,C). In addition, one conventional hydrogen bond is predicted between the carbonyl oxygen of carvotanacetone and Trp162 (Figure 12B,C), which may contribute to the stabilisation of the docked pose. The predicted binding mode of D-limonene is shown in Figure 12D–F. The ligand is accommodated within a hydrophobic region of the Hsp90α binding pocket (Figure 12D). The interaction pattern is dominated by van der Waals, alkyl and π-alkyl contacts with neighbouring residues (Figure 12E,F), whereas no conventional hydrogen bonds were observed. This interaction profile is consistent with the non-polar character of D-limonene.

## 3. Discussion

The hydroalcoholic *A. graveolens* seed extracts contained predominantly polar phenolic constituents, while the EO consisted mainly of volatile lipophilic monoterpenes. However, one must also consider that in products of vegetal origin, such as extracts and essential oils, complex additive, synergistic or antagonic interactions may occur even between minor components; therefore, the identified major components are most likely to have been responsible for the respective biological effects but this hypothesis was not definitively proven.

The LC-MS analysis revealed a profile dominated by hydroxycinnamic acid including chlorogenic, caffeic, *p*-coumaric, ferulic and sinapic acids, in agreement with previous studies that describe *A. graveolens* L. as a natural source of hydroxycinnamic derivatives and other phenolic compounds [29,30]. Additionally, chlorogenic, ferulic and sinapic acids have previously been identified in *A. graveolens* leaves, with hydroethanolic extraction conditions strongly influencing their recovery [19]. Among the analysed extracts, A2 showed the highest total content of polyphenolic compounds quantified by LC-MS, while A4 revealed the highest TPC, TFC and CTC values. This apparent discrepancy reflects the different analytical principles of the applied methods. LC-MS provides compound-specific information and, in the present study, quantified only phytochemicals included in the targeted analytical method. However, the Folin–Ciocalteu, aluminium chloride, and vanillin–HCl assays provide global estimates based on chemical reactivity and may also respond to constituents other than those specifically quantified by LC-MS. Therefore, the higher spectrophotometric values of A4 may reflect the contribution of additional reducing, aluminium-reactive or vanillin-reactive compounds. Conversely, A2 exhibited higher amounts of several monitored constituents, particularly fisetin, ferulic acid and p-coumaric acid. Jevtovic et al. [19] reported chlorogenic, ferulic and sinapic acid concentrations of approximately 103.75, 6.05 and 2.19 μg/g plant material, respectively, following the optimised extraction of *A. graveolens* leaves with 50% ethanol. In the present study, the highest concentrations were 1.99 μg/mg dry extract for chlorogenic acid in A4, 2.08 μg/mg dry extract for ferulic acid in A2 and 3.27 μg/mg dry extract for sinapic acid in A3. Direct quantitative comparison between these datasets is not appropriate because results are expressed as dry material versus dry extract, and also, different plant organs were analysed. The TPC values range between 169.4 ± 0.5 and 177.3 ± 0.5 mg GAE/g extract were higher than those previously reported for *A. graveolens* preparations, including values of 7.06–19.09 mg GAE/g dry extract [31] and 23.35–52.65 mg GAE/g extract [32]. Such differences emphasize the influence of the extraction procedure, solvent composition and analytical approach on the reported phytochemical content. Therefore, spectrophotometric indices should be interpreted as global estimates of chemical reactivity rather than absolute concentrations of individual phenolic compounds. Similarly, the higher TFC and CTC values observed for A4 indicate a greater response toward aluminium chloride and vanillin–HCl, respectively, without necessarily implying higher concentrations of the individual flavonoids or tannins quantified by target analysis [32].

The GC-MS analysis of AGEO revealed a completely different chemical composition compared to the hydroalcoholic extracts, with carvotanacetone (60.41%) and limonene (38.70%) as the dominant constituents. Previous studies have shown variability in AGEO composition; Ozliman et al. [14] reported 10.23–20.05% limonene and 5.04–21.76% carvotanacetone, while Jirovetz et al. [33] identified D-carvone (50.1%) and D-limonene (44.1%) as major constituents. Stanojevic et al. [34] and Milenkovic et al. [15] also reported dill seed oils rich in carvone, with Milenkovic et al. reporting 46.1–49.8% carvone and 37.8–43.8% limonene. The higher carvotanacetone percentage reported in the current study may therefore indicate a specific chemotype of the plant.

The distinct chemical composition of the extracts and the EO is important when interpreting the in vitro effects. The hydroalcoholic extracts contained mainly phenolic acids, flavonoids and tannins displaying polar character, able to act through redox reactions, metal chelation and interaction with proteins. Conversely, the EO mainly contained lipophilic monoterpenes, able to interact with the components of biological membranes and intracellular lipids. Previous literature findings indicate that *A. graveolens* L. extracts and EO can function as antioxidant and antibacterial agents; also, they may inhibit enzyme activity but these effects are strongly influenced by the extraction procedure and the extract composition [35,36,37,38]. These chemical differences support comparative interpretation of the biological results observed in the present study. A2 revealed the highest content of phenolic compounds identified by LC-MS, while A4 exhibited the highest TPC, TFC and CTC values. In contrast, the EO displayed a composition rich in monoterpenoids, particularly, carvotanacetone and limonene, which indicates that any in vitro difference compared to the hydroalcoholic extracts should be assessed as the result of distinct phytochemical profiles rather than as quantitative differences within the same class of compounds.

As mentioned previously, both AGEO and *A. graveolens* L. extracts have shown promising cytotoxic effects in multiple cell lines [20,22,23,24]. To the best of our knowledge, this is the first study to evaluate the cytotoxic activity of *A. graveolens* EO and extracts against A375, SK-OV-3 and PANC-1 cancerous cells. These cell lines were selected in order to provide a panel of human malignant cells with distinct tissue origin and molecular characteristics, which may allow determination of whether the biological effects of *A. graveolens* products are restricted to a particular cancer type or can be inflicted in different tumour cells. A375 cells stand as a well-established BRAF-mutated melanoma model where mitochondrial metabolic reprogramming resulted in increased cell survival as well as drug resistance; notably, the disruption of mitochondrial function was proven to suppress A375 cell viability [39]. PANC-1 cells are widely used as a model for poorly differentiated pancreatic adenocarcinoma; they display molecular alterations such as the activation of the KRAS-related signalling pathway and the disruption of several major tumour-suppressor pathways, making them particularly significant in the assessment of potential agents against highly aggressive, therapeutically challenging tumours [40]. SK-OV-3 cells were included as an ovarian carcinoma model in order to extend the current analysis to a gynecological malignancy; SK-OV-3 cells are among the most frequently employed ovarian cancer cell lines in experimental studies [41]. Moreover, mitochondrial alterations and the implication of proteins associated with oxidative phosphorylation have been directly associated with SK-OV-3 cells’ response to cytotoxic treatment [42].

AGEO and A1–4 showed a dose-dependent cytotoxic activity, although the strongest effect was observed in the case of AGEO. This apparently greater cytotoxic activity of AGEO should be interpreted with caution, because the tested concentrations of the liquid EO were expressed as volume percentages, and as such, they cannot be directly compared with the concentrations used for dry extracts, expressed as mass concentrations. Furthermore, AGEO represents a concentrated lipophilic mixture dominated by volatile monoterpenes, whereas the hydroethanolic extracts contain a complex polar matrix in which the identified phenolics account for only part of the total dry mass. The stronger effect of AGEO may therefore reflect both its chemical composition and the ability of lipophilic monoterpenes to partition rapidly into cellular and mitochondrial membranes, rather than a direct quantitative superiority over the extracts.

Similar findings have been reported in studies that simultaneously tested plant extracts and EOs. For example, EO of *Cyperus longus* showed lower IC_50_ values against MCF-7 and PC3 cells compared to methanolic extract, aqueous, dichloromethane and ethyl acetate fractions, indicating a more powerful cytotoxic effect. In addition, this study highlighted the variations in bioactivity dependent on the nature of the extract/fraction and solvent used [43]. Based on these observations and in concordance with chemical composition analysis, the activity of different plant products is correlated with their chemical profile and varies based on the preparation process. Also, studies on EO’s individual components should be taken into consideration. For example, limonene, one of the major constituents of AGEO, has the ability to reduce the viability of A375 cells in a dose-dependent manner [44]. It also showed effects against pancreatic cancer both in vitro and in vivo [45]. Although carvotanacetone, AGEO’s main constituent, has not yet been extensively analysed for its cytotoxic effects, several papers report the cytotoxic potential of its derivatives against human melanoma, SK-MEL cells, and ovarian cancer, SK-OV-3 cells [46]. Similarly, it was reported that overall, *A. graveolens* essential oil exhibited cytotoxic effects in HepG2 cells in a concentration-dependent manner by promoting G2/M and pre-G1 cell cycle arrest together with apoptotic cell death [23].

Regarding the *A. graveolens* extracts, the most pronounced effects in all cancer types were exhibited by A2 and A4, which could also be linked to their chemical composition. Our findings are in line with previous studies that reported the cytotoxic activity of *A. graveolens* products. For example, a methanolic dill extract was reported to exert dose-dependent cytotoxic effects in MCF-7, A549 and HeLa cells [20]; similarly, Mohammed et al. revealed that an ethyl acetate extract fraction of dill seeds was able to inhibit HepG2 cell proliferation [47].

Some of the compounds detected in higher concentrations in the extracts have demonstrated cytotoxic effects on their own in multiple studies. For instance, proanthocyanidins or condensed tannins have shown favourable effects in colon cancer, oral squamous cell carcinoma and breast cancer [48]. Furthermore, Syed et al. observed that fisetin induced apoptotic cell death when tested on A375 melanoma cells at a concentration of 60 μM, correlating this effect with endoplasmic reticulum stress induction [49]. Similarly, p-coumaric acid significantly inhibited the proliferation of A375 cells by downregulating Bcl-2, upregulating Bax and Apaf1, increasing cytochrome c levels and cleaving caspase-3 and caspase 9, thus leading to apoptosis. Along with this mechanism, p-coumaric acid also downregulated CDK2 and Cyclin A cell cycle proteins, ultimately leading to cell cycle arrest in phase S [50]. These observations are in concordance with the results of the morphological analysis, where treatment with A1–4 extracts induced morphological changes that could indicate apoptosis in A375, but also in SK-OV-3 and PANC cell lines. However, additional experiments are needed in order to confirm the proposed mechanism.

Regarding the impact of *A. graveolens* L. products on healthy cells, although high doses of A1–4 and AGEO led to a significant decrease in HaCaT cell viability, the respective effect was still less pronounced than in the cancer lines, indicating a certain level of selectivity. The respective selectivity could be explained by different metabolic activity of the healthy cells compared to cancer cells [51]. Further analysis of the possible mechanisms responsible for *A. graveolens* L. cytotoxic activity revealed reduced OXPHOS efficiency. Oxidative phosphorylation, or OXPHOS, has an important role in cancerous cells, as recent studies have revealed its implication in cancer proliferation, survival and metastasis [52]. Although both aerobic glycolysis and oxidative phosphorylation are active in cancerous cells due to metabolic reprogramming, both slow-cycling and actively proliferating tumour cells utilise oxidative phosphorylation [51]. Furthermore, cancer cells that develop resistance to treatment can display elevated OXPHOS activity [53]. Therefore, drugs that target and inhibit OXPHOS represent a new research area in cancer treatment [52,53]. In the case of AGEO and A2 and A4 extracts, we observed a reduction in the oxidative phosphorylation derived from Complex I (OXPHOS CI) and from convergent complexes I and II (OXPHOS CI+II). However, as there was an increase in ETC CI and ETC CI+II respiratory states, the OXPHOS reduction should not be attributed to the inhibition of complexes I and II. Another observed effect that can also be linked to decreased OXPHOS, is the mitochondrial uncoupling-like effect [51]. Mitochondrial uncoupling is described as a dissociation between the generation of MMP and its use for ATP transformation [54]. The process is linked to an increase in proton leak across the inner mitochondrial membrane [55], leading to an increase of State 2 and State 4 [51]. Regarding the relationship between mitochondrial uncoupling and its role in cancer, it has been suggested that it is a complex one, resulting in the promotion of cancer cell survival in some cases and limiting tumour progression in others [56]. However, plant-derived compounds such as guttiferone A and nemorosone have been identified as mitochondrial uncouplers, while also showing cytotoxic effects [57]. One important characteristic of mitochondrial uncouplers is their ability to cause a loss of MMP [57]. Hence, determination of MMP was one of the natural directions for this research, especially due to its connection to apoptosis. In any case, the concentration of A2 and A4 extracts at which the mitochondrial activity was determined was that which showed significant cytotoxic effects in the cell viability assay; in fact, the tested dose (720 μg/mL) was the closest to the reached IC_50_ values, which is why it was selected for the mechanistic experiments. This use of higher concentrations, such as 720 μg/mL, is not an isolated case with regard to plant extracts, including dill extracts. For example, there are reports of alterations in mitochondrial function at even higher doses, such as 1000 μg/mL of *A. graveolens* methanolic extract in the experiments conducted by Oqail et al. [20]. In this context, the literature reports a range of concentrations for various *A. graveolens* extracts. Mohammed et al. calculated an IC_50_ of 0.393 mg/mL for HepG2 cells in the case of ethyl acetate extract fraction of dill [47], while Al-Oqail et al. reported three different values of IC_50_ for specific cell lines, given by *A. graveolens* methanolic extract: IC_50_ of 104 μg/mL for MCF-7, 122 μg/mL for A-549, and 156 μg/mL for HeLa cells [20]. Nevertheless, it should be mentioned that the chemical composition of every extract differs due to different methods of extraction, and therefore, the tested concentrations cannot be precisely compared. Likewise, it was observed that every cell line responded differently to the applied treatment. However, in order to further clarify the exact causes responsible for cellular effects and to clearly distinguish the pharmacological mechanisms from high-dose toxicity, a more detailed study of mitochondrial activity should be performed at lower doses to investigate the dose–response relationship. The rationale behind the decision to investigate cancer cell bioenergetics arises from previously reported evidence indicating that *A. graveolens* extracts and several compounds in their composition are able to interfere with mitochondrial function. As an example, an ethyl acetate extract fraction of *A. graveolens* seeds was revealed to alter the mitochondrial membrane potential in HepG2 cells, in addition to producing changes in ROS concentration and the simultaneous activation of caspases 9 and 3/7, thus confirming that its biological activity involves the intrinsic mitochondrial apoptotic pathway [47]. Similarly, mechanistic studies in MCF-7 cells indicated that the *A. graveolens* methanolic extract increased ROS levels, altered the mitochondrial membrane potential and activated caspases-3 and -9 [20]. Moreover, D-limonene was proven to induce intrinsic apoptosis in LS174T colon cancer cells, as revealed by an increased Bax/Bcl-2 ratio, cytochrome c release and caspase-9/caspase-3 activation [58]. Of note, a direct effect on cancer cell bioenergetics has also been reported for the flavonoid quercetin, which was identified in *A. graveolens* extracts; in melanoma cells, quercetin was able to inhibit the mitochondrial respiration in a concentration- and time-dependent manner, including basal and maximal respiration, oxygen consumption and respiratory reserve capacity while also decreasing glycolytic activity [59]. Collectively, these findings suggested the hypothesis that the disruption of the mitochondrial energy metabolism may stand as one underlying molecular mechanism responsible for the observed cytotoxic effects. Nevertheless, plant extracts and EO are complex mixtures of individual components whose biological effects cannot be directly extrapolated to the whole product; instead, the overall biological activity may be the result of additive or synergistic interactions among multiple bioactive individual components.

The network pharmacology analysis suggests that AGEO might exert its toxicity in pancreatic cancer, one of the most sensitive cell lines in the cytotoxicity screening, through a multitarget approach. The compound–target network indicated that AGEO constituents could exert their effect through common targets, such as PTGS2, PPARG, TLR4 and HSP90AA1, while β-myrcene and carvotanacetone might modulate unique targets such as MDM2 and STAT3, thus potentially expanding the biological effect of the AGEO. The synergistic effect implied aligns with the polypharmacology concept of medicinal plants, which states that extracts exert a superior effect compared to their individual components [60]; however, studies comparing the individual components within AGEO are required to verify this hypothesis. Furthermore, the protein–protein interaction network revealed STAT3 and HSP90AA1 as potential targets that might be responsible for the cytotoxicity of AGEO in the PANC-1 cell line. Thus, HSP90α protein encoded by *HSP90AA1* was selected for molecular docking due to the predicted interaction with all the AGEO constituents detected. HSP90α is a promising target for drug development as it plays a regulatory role in cancer-related mechanisms that promote proliferation, survival and resistance to treatment [61]. Pan-HSP90 inhibitors such as ganetespib and tanespimycin, a geldanamycin derivative, have been investigated in preclinical and clinical trials [62,63]. The selection of Hsp90α is also relevant to the biological effects observed in the present study. AGEO induced pronounced cytotoxic effects in PANC-1 cells, together with mitochondrial membrane depolarization, altered mitochondrial coupling efficiency and morphological changes consistent with apoptosis. In this context, the identification of HSP90AA1 as a common predicted target of the AGEO constituents provides a potential mechanistic link between the network pharmacology findings and the experimentally observed cellular effects. However, the direct involvement of Hsp90α in these effects was not experimentally demonstrated in the present study. Molecular docking predicted that all six volatile constituents identified in AGEO could be accommodated within the ATP-binding pocket of Hsp90α, although with different predicted binding affinities. Carvotanacetone and D-limonene exhibited the most favourable docking scores among the investigated phytochemicals, which is noteworthy considering that these two compounds account for almost the entire volatile fraction of the AGEO. A more detailed analysis of the predicted binding modes revealed that carvotanacetone established predominantly hydrophobic interactions together with a conventional hydrogen bond involving Trp162, whereas D-limonene interacted almost exclusively through hydrophobic contacts, reflecting its non-polar chemical structure. Although molecular docking is a predictive computational approach, the favourable binding poses and interaction profiles observed for the major constituents of AGEO support the hypothesis that Hsp90α may represent a molecular target involved in the biological activity of the EO.

The enrichment analysis highlighted the role of identified targets for AGEO in the regulation of the apoptotic signalling pathway (GO:2001233) and the extrinsic apoptotic pathway (GO: 2001236), suggesting that AGEO might modulate apoptotic signalling. These findings are consistent with the immunofluorescence results showing the ability of AGEO to induce apoptotic-like morphological changes in the PANC-1 cell line. Nevertheless, the computational analyses can provide only a predictive assessment that requires future experimental validation.

A noteworthy limitation of the present study is the variable chemical composition of the samples that can be significantly dependent on the plant origin and developmental stage, harvesting, processing and storage conditions, thus limiting the extrapolation of the current results to other *A. graveolens* preparations.

## 4. Materials and Methods

### 4.1. Chemicals, Reagents, Standards and Equipment

Acetic acid 99.9%, methanol and ethanol were purchased from Merck (Darmstadt, Germany). Concentrated hydrochloric acid, vanillin, potassium acetate, aluminium chloride, quercetin, sodium carbonate, Folin–Ciocalteu reagent and gallic acid were obtained from Sigma-Aldrich Chemie GmbH (Taufkirchen, Germany). Apigenin, hyperoside, kaempferol, luteolin, rutin, quercetol, quercetin, fisetin, chlorogenic acid, caffeic acid, rosmarinic acid, caftaric acid, gentisic acid, p-coumaric acid, ferulic acid, sinapic acid, isoquercitrin and myrcetin, were used as reference standards for LC-MS and were purchased from Sigma-Aldrich Chemie GmbH (Taufkirchen, Germany). Ultrapure deionized water was obtained with a Milli-Q^®^ Integral Water Purification System (Merckmilipore, Darmstadt, Germany) and used for preparation of the sample solutions.

The experimental procedures were carried out using a Shimadzu UV-1900i UV-Vis spectrophotometer (Shimadzu Scientific Instruments Inc., Columbia, MD, USA), operating at a wavelength between 400–800 nm. Other equipment included an electrical grinder, a vacuum filtration system, a Buchner funnel, a Soxhlet installation, a Craveiro-type apparatus, a rotary evaporator and a vortex mixer.

### 4.2. Plant Material

A single batch of dried *A. graveolens* seeds were received as a donation from “King Michael I” University of Life Sciences (Timișoara Romania Herbarium, voucher specimen number VSNH.BUASTM—129). The seeds were collected in Timis county in September 2024, at the optimal harvest point when the seeds on the umbel were light brown, and then dried in the dark at 24 °C. Botanical authentication was carried out by specialists at the donating institution. The same batch of plant material was used to prepare all the extracts and essential oil investigated in the current study. Before further processing, the dried *A. graveolens* seeds were pulverized using an electrical grinder.

#### 4.2.1. Preparation of Hydroethanolic Extracts

The two methods used, maceration and Soxhlet extraction, were selected as conventional solid–liquid extraction methods under different conditions. The 40% and 60% (*v*/*v*) ethanol mixtures were chosen to evaluate the solvent composition around the 50% ethanol range previously reported as suitable for the recovery of the phenolic compounds [19]. Soxhlet extraction with hydroalcoholic solvent has previously been applied to *A. graveolens* seeds for the recovery of the phenolic compounds [32].

Maceration was performed in the dark at room temperature for 10 days, and Soxhlet extraction was carried out for 15 full cycles. The ratio between the dry pulverized material and solvent was 1:10 (*w*/*v*) for maceration and 1:13 (*w*/*v*) for Soxhlet. In detail, 23 g of finely ground plant material was extracted with 230 mL solvent in the case of maceration. For Soxhlet extraction, 23 g of plant material was extracted with 300 mL. The sample codes, extraction methods and corresponding solvents are presented in Table 1. Following maceration, extracts A1 and A2 were separated from the residual plant material by vacuum filtration using a Buchner funnel fitted with filter paper. Extracts A3 and A4, obtained by the Soxhlet method, were collected from the extraction flask. The solvent was removed entirely from all the extracts using a rotary evaporator (reduced pressure), at a temperature of 60 °C. The resulting dry extracts were stored in the dark at 4 °C pending further analysis.

#### 4.2.2. *A. graveolens* L. EO Extraction

Steam hydrodistillation was selected as a conventional method for the isolation of the volatile fraction of *A. graveolens* seeds, as previously reported for dill essential oil extraction [6]. The extraction was performed for 4 h at 100 °C using a previously described Craveiro-type apparatus [64,65] The distillation rate was not specifically monitored or quantified. However, continuous steam generation under steady boiling conditions was maintained throughout the extraction procedure. Briefly, steam was generated by heating water in a 3000 mL glass boiler equipped with an electrical resistance, continuously refilling with water whenever it was necessary. The steam was guided to the bottom of a 1000 mL glass extraction vessel that contained the pulverised plant material on a perforated plate positioned a few centimetres above the extraction chamber base. The volatilised EO constituents and steam were subsequently condensed with a water-cooling system. To minimize the formation of thermal degradation artefacts, the EO and hydrosol were collected in a 250 mL glass receiver fitted with a water-cooling jacket and a hydrosol overflow outlet. After separation of the phases, the obtained EO was dried over anhydrous sodium sulphate to remove residual water and stored in sealed vials at −18 °C until further analysis.

The extraction yield was calculated using the following equation: oil weight/dried plant weight × 100.

Each extraction described in Section 4.2.1 and Section 4.2.2 was performed once, and the resulting preparations were further used for the phytochemical and biological evaluation.

### 4.3. LC-MS Instrumentation and Analytical Conditions

High-performance liquid chromatography (HPLC/LC) coupled with mass spectrometry (MS) experiments were conducted on a 6120 LC-MS analytical system from Agilent (Santa Clara, CA, USA) consisting of 1260 Infinity HPLC equipped with a G1322A degasser, G1311B quaternary pump, G1316A column thermostat, G1365C MWD detector and G7129A autosampler coupled with a quadrupolar (Q) mass spectrometer equipped with an electrospray ionization source (ESI). The LC-MS system was connected to a PC computer running the OpenLAB CDS ChemStation Workstation software, version C.01.08 to control the instrument and to acquire and process LC-MS data.

Screening and quantification of polyphenolic compounds in *A. graveolens* seed extract samples A1–A4 were conducted by LC-MS. Polyphenols were separated on a reverse-phase Zorbax Eclipse Plus C18 column (3.0 × 100 mm × 3.5 µm) by an LC-MS method that enabled the screening and quantification of analysed extracts for 18 polyphenols including rosmarinic acid, caftaric acid, gentisic acid, chlorogenic acid, caffeic acid, p-coumaric acid, ferulic acid and sinapic acid, hyperoside, isoquercitrin, rutin, myricetin, fisetin, quercitrin, quercetol, luteolin, kaempferol and apigenin, as described previously [66,67]. Polyphenolic compounds were screened in gradient elution with a mobile phase that consisted of a mixture of 0.1% acetic acid solution and methanol as follows: 5 min 5% methanol, up to 38 min in gradient elution reaching 42% methanol, and 5% methanol up to 42 min. The elution of all components was completed in about 40 min at a flow rate of 1 mL/min, injection volume 10 µL and column temperature of 40 °C. UV detection of polyphenols was conducted at 330 and 370 nm. MS detection was achieved by electrospray ionization (ESI) in the negative ion mode using single ion monitoring (SIM) that enabled the simultaneous screening and quantification of all 18 screened compounds. MS parameters were capillary voltage 3500 V, dry gas flow 12 L/min at 350 °C, nebuliser pressure 55 psig and fragmentor at 70. For the quantification of phytocompounds in the extracts, calibration curves were conducted by the external standard method in the 0.05–2 µg/mL range, for a six-point plot for each compound [33]. The *m*/*z* scale of the mass spectrum was calibrated using an external calibration standard ESI Tuning Mix from Agilent (Santa Clara, CA, USA).

#### Sample Preparation for LC-MS Analysis

All dry extract samples were dissolved in pure methanol, homogenized with a WisdVM-10vortex mixer (WitegLabortechnik, Wertheim, Germany) and centrifuged for 2 min at 10,000 rpm in a ThermoMicro CL17 microcentrifuge (Thermo Fisher Scientific, Waltham, MA, USA). The supernatant was collected and submitted to LC-MS analysis.

### 4.4. GC-MS Analysis of AGEO

AGEO was analysed by gas chromatography–mass spectrometry (GC–MS) using an Agilent 6890 GC system coupled with an Agilent 5973 MSD quadrupole mass spectrometer (Agilent Technologies, Santa Clara, CA, USA). Separation of analytes was performed on a VF-5MS capillary column (30 m × 0.25 mm i.d., 0.25 µm film thickness), employing helium as the carrier gas at a constant flow rate of 1 mL/min. The oven temperature program was set from 50 °C to 250 °C, increasing at 6 °C/min. Before analysis, concentrated oil samples were diluted 1:1000 in hexane and injected into the system. Sample injection was performed in splitless mode, with the injector temperature set to 230 °C. A 4 min solvent delay was applied before mass spectral acquisition. The ion source temperature was set to 150 °C, and electron ionization was performed at 70 eV. Mass spectra were recorded in full-scan mode over an *m*/*z* range of *m*/*z* 50–600. Compound identification was achieved by comparing the experimental spectra with entries from the NIST11 mass spectral library using ChemStation software version B.01.00 (Agilent Technologies, Palo Alto, CA, USA). Retention indices (RI_calc_) were calculated based on a homologous series of C_8_–C_20_ n-alkanes (Sigma-Aldrich, Germany) and subsequently compared with literature retention indices (RI_lit_) obtained from the NIST Chemistry WebBook (National Institute of Standards and Technology, Gaithersburg, MD, USA), determined under comparable chromatographic conditions using the same stationary phase [68]. Authentic reference standards were not used for compound identification and quantification. The reported GC-MS results represent the relative peak area percentages, calculated by dividing each peak area by the total integrated area of all detected peaks. Peak integration skipped those minor peaks with a signal-to-noise (S/N) ratio of 10:1. To ensure accuracy and eliminate contamination risks, solvent blanks were analysed under identical GC-MS conditions.

### 4.5. Spectrophotometric Assay

The values of the absorbance for the spectrophotometric assays were obtained using a Shimadzu UV-1900i UV-Vis spectrophotometer.

#### 4.5.1. Total Phenolic Content (TPC)

The TPC of the extracts was assessed by Folin–Ciocalteu colorimetric assay with minor adaptations of a previously described protocol [69]. For the analysis, 0.5 mL of either gallic acid standard or sample solution was combined with 2.5 mL of Folin–Ciocalteu reagent diluted tenfold with distilled water. After 5 min at 25 °C, 2.0 mL of 7.5% sodium carbonate solution was added. The reaction mixtures were homogenised by vigorous vortexing and maintained for 30 min at 25 °C under light-protected conditions. The absorbance was recorded at 765 nm using the appropriate blank as reference. Since the extracts displayed a slight background colouration, an absorbance correction step was included. To verify whether the hydroethanolic composition influenced the response, gallic acid calibration curves were first generated separately in 40% and 60% ethanol. Because both calibration plots showed similar linear behaviour and regression characteristics, one calibration equation was used for the quantification of phenolic compounds in all samples. TPC values are reported as milligrams of gallic acid equivalents per gram of dry extract (mg GAE/g extract).

#### 4.5.2. Total Flavonoid Content (TFC)

TFC was calculated by employing a quercetin standard and the method reported by Chang et al. [70] slightly modified. In this process, 0.5 mL quercetin standard or extract solution was added to 1.5 mL ethyl alcohol. Afterwards, 0.1 mL aluminium chloride of concentration 10%, 0.1 mL potassium acetate and 2.8 mL water were added. The resulting mix was then intensely vortexed and incubated at room temperature (25 °C) in the dark, for half an hour. Next, the value of the absorbance was measured at 415 nm against the blank. Subsequently, like for the TPC assay, an absorbance correction was applied to eliminate the absorbance generated by the extract colour. To minimize solvent-related variations, the dry extracts and the quercetin standard were dissolved in 80% ethanol. TFC values were expressed as mg quercetin equivalents per g dry extract (mg QE/g extract).

#### 4.5.3. Condensed Tannin Content (CTC)

CTC was determined using the acidified vanillin method, according to the original method [71] and most recently described by Géorcelin et al. [72]. Briefly, 0.5 mL catechin standard solution or extract solution was mixed with 3.0 mL of vanillin (concentration 4%) in methanol and 1.5 mL concentrated hydrochloric acid. The resulting mixture was then thoroughly vortexed and incubated for 15 min at 25 °C, in the dark. Afterwards, the absorbance was measured at 500 nm against the corresponding blank. Like in the previous assays, a correction of the absorbance was realized. The catechin standard and dry extracts were dissolved in 80% ethanol to ensure similar solvent conditions and to minimize solvent-induced variations. CTC values were expressed as mg catechin equivalents per g dry extract (mg CE/g extract).

### 4.6. Cell Culture

For the in vitro evaluations, several human cell lines were used, including immortalised keratinocytes (HaCaT) and tumour cell lines represented by melanoma (A375), pancreatic adenocarcinoma (PANC-1), and ovarian adenocarcinoma (SK-OV-3). The HaCaT cell line was obtained from CLS Cell Lines Service GmbH (Eppelheim, Germany), while the tumour cell lines were purchased from the American Type Culture Collection (ATCC, Lomianki, Poland). Cells were stored in liquid nitrogen until use and subsequently cultured under standard conditions at 37 °C in a humidified atmosphere containing 5% CO_2_. The culture medium used for HaCaT, A375 and PANC-1 was high-glucose Dulbecco’s Modified Eagle Medium (DMEM) and McCoy’s 5A medium for SK-OV-3, all supplemented with 10% fetal bovine serum and 1% penicillin–streptomycin.

### 4.7. Cell Viability Assay

Cell viability was assessed using the Alamar Blue assay following a 24 h treatment with A1–A4 (3.6, 36, 360, 720 and 1000 μg/mL), as well as AGEO (0.01%, 0.05% and 0.1%). Cells were seeded in 96-well plates at a density of 1 × 10^4^ cells/well and cultured until reaching 80–85% confluence. After attachment, the culture medium was replaced with fresh medium containing the plant extracts or the EO. After 24 h treatment, Alamar Blue (0.01%) was added to each well and incubated at 37 °C, in the dark, for 3 h. Fluorescence was measured at 528 nm excitation and 590 nm emission using a Synergy HTX microplate reader (BioTek Instruments, Inc., Winooski, VT, USA). All experiments were performed in biological and technical triplicate, and results were expressed relative to untreated control cells (100% viability).

### 4.8. Immunofluorescence Assay

Immunofluorescence analysis was performed to evaluate the cytoskeletal and nuclear alterations induced by treatment with A1–A4 at a concentration of 720 μg/mL and AGEO at concentrations of 0.05%. Cells were seeded in 12-well plates at a density of 2 × 10^5^ cells/well and cultured until reaching 80–85% confluence. After 24 h of treatment, cells were washed with phosphate buffer saline, fixed with 4% paraformaldehyde for 10 min, and permeabilised with 0.1% Triton X-100 for 15 min. Blocking of non-specific binding sites was performed using 3% bovine serum albumin for 30 min. For cytoskeletal visualization, cells were incubated with a mouse monoclonal anti-β-actin antibody (Product #MA1-140, Thermo Fisher Scientific, Inc., Waltham, MA, USA; 1:2000) for 1 h at room temperature, followed by incubation with an Alexa Fluor Plus 488-conjugated goat anti-mouse IgG (H+L) secondary antibody (Product #A32723, Thermo Fisher Scientific, Inc., Waltham, MA, USA; 1:500) for 30 min in the dark. Nuclei were counterstained with Hoechst 33342 (Product #62249, Thermo Fisher Scientific, Inc., Waltham, MA, USA, 1:2000) for 10 min under light-protected conditions. Samples were subsequently visualised using a Thermo Scientific EVOS™ M5000 Imaging System (Thermo Fisher Scientific, Inc., Waltham, MA, USA); the images were analysed with a 40× objective to assess cytoskeletal integrity and nuclear morphology. All experiments were performed in biological and technical triplicate.

### 4.9. High-Resolution Respirometry

High-resolution respirometry was used to assess mitochondrial respiratory function. The experiments were performed using an Oxygraph-2k system (Oroboros Instruments, Innsbruck, Austria), following the substrate–uncoupler–inhibitor titration protocol described by Petrus et al. [73]. Oxygen flux was recorded using DatLab software 4 (Oroboros Instruments, Innsbruck, Austria).

Initially, A375, PANC-1 and SK-OV-3 cell lines were cultured in T75 flasks and were trypsinised after reaching 85% confluency. An adequate number of cells (2 × 10^6^ cells/chamber) was suspended in mitochondrial respiration medium MIRO5 (110 mM D-sucrose, 20 mM taurine, 0.5 mM EGTA, 20 mM HEPES, 3 mM MgCl_2_, 60 mM lactobionic acid, 10 mM KH_2_PO_4_ and 1 g/L bovine serum albumin; pH was adjusted to 7.1 using KOH) and introduced into oxygraph chambers. Prior to cell addition, the instrument was calibrated and A2 (720 μg/mL), A4 (720 μg/mL) and AGEO 0.05% were inserted into the test chamber. After 10 min of equilibration in which the routine respiration was established, digitonin (1 μg/L × 10^6^ platelets) was added in order to permeabilise the cell membrane. Subsequently, glutamate (5 mM) and malate (5 mM) were added, leading to State 2CI or Complex I-linked respiration assessment. The following step included the addition of ADP (1 mM), causing a stimulation of oxidative phosphorylation dependent on Complex I (OXPHOSCI). Afterwards, succinate (10 mM) was added, activating complex II and ensuring the maximal oxidative phosphorylation, dependent on both complexes I and II (OXPHOSCI+II). Complex V was then inhibited by the addition of oligomycin (1 µg/mL), and State 4CI+II or non-phosphorylating respiration state was measured. Following a stepwise titration of FCCP (1 µM/step), maximal uncoupled respiration or maximal capacity of the electron transport system (ETSCI+II) was reached. Subsequently, rotenone (2 µM) was added in order to inhibit complex I and assess the electron transport capacity dependent on Complex II (ETSCII). Finally, antimycin A (1 µg/mL) was added, after which Complex III was inhibited and residual oxygen consumption was measured. All experiments were conducted in biological triplicate.

### 4.10. Assessment of the Mitochondrial Membrane Potential

The mitochondrial membrane potential (MMP) was assessed using the JC-1 kit (JC1- Mitochondrial Membrane Potential Assay Kit ab113850, Abcam, Cambridge, MA, USA), according to the manufacturer’s specifications [74]. Briefly, after reaching approximately 85% confluency, A375, PANC-1 and SK-OV-3 cells were treated for 24 h with A2 (720 μg/mL), A4 (720 μg/mL) and AGEO 0.05%. The cells treated with 50 μM carbonyl cyanide 3-chlorophenylhydrazone FCCP were used as a positive control for mitochondrial depolarization. Following the treatment period, 2 µM of JC-1 was added and the plates were incubated for 30 min at 37 °C in the dark. Fluorescence was measured (Ex/Em: 535/590 nm for JC-1 aggregates and 475/530 nm for JC-1 monomers) using a Synergy HTX microplate reader (BioTek Instruments, Inc., Winooski, VT, USA). Mitochondrial membrane potential was evaluated based on the red-to-green fluorescence ratio. All experiments were performed in biological and technical triplicate.

### 4.11. Statistical Analysis

The cell viability results were analysed using one-way analysis of variance (ANOVA), followed by Dunnett’s multiple comparisons test. For the high-resolution respirometry studies, the statistical differences vs. control were determined using two-way ANOVA with Bonferroni’s multiple-comparison post-test. Values were considered to be statistically significant if *p* < 0.05 (* *p* < 0.05; ** *p* < 0.01; *** *p* < 0.001 vs. control cells).

### 4.12. Network Pharmacology

#### 4.12.1. Gene Selection

The top 500 protein-coding genes for pancreatic cancer were retrieved from the GeneCards database based on the relevance score [75]. Then, to facilitate the next steps, the genes were converted into their corresponding protein-coding entries using the UniProt database [76].

#### 4.12.2. Ligand Preparation

The tridimensional structures and SMILES of the six compounds identified in AGEO through GC-MS, namely, α-pinene (PubChem ID: 6654), β-thujene (PubChem ID: 520384), β-myrcene (PubChem ID: 31253), 3-carene (PubChem ID: 26049), D-limonene (PubChem ID: 440917) and carvotanacetone (PubChem ID: 6432475) were downloaded from PubChem [77], and then underwent geometric optimization under the MMFF94 forcefield in Avogadro (v. 2.0) [78], to ensure an appropriate tridimensional conformation.

#### 4.12.3. In Silico Target Prediction

The SMILES codes of the compounds were imported into Swiss Target Prediction [79] to predict relevant biological targets. Moreover, their tridimensional structures were imported into the PharmMapper server [80] to increase the number of predicted targets. In PharmMapper, the maximum number of targets was set to 500 within the druggable pharmacophore models category. Lastly, the intersection of the predicted targets with the proteins encoded by genes relevant for pancreatic cancer was made using the Interactivenn web tool [81].

#### 4.12.4. Compound–Target Network Construction

A compound–target network was constructed using Cytoscape (v3.10.4) [82] to visualise the interactions between the six compounds identified by GC–MS in AGEO and their predicted pancreatic cancer-related protein targets. In the network, oval nodes represented the AGEO constituents, whereas the rectangular nodes represented predicted protein targets. Additionally, edges depicted predicted interactions between the compounds and target proteins, either by Swiss Target Prediction or PharmMapper.

#### 4.12.5. Protein–Protein Interaction Network and Enrichment Analysis

To identify relevant biological interactions, the common protein targets of the AGEO compounds and pancreatic cancer were imported into the STRING database [83]. The analysis had a confidence level greater than 0.700 and a false discovery rate (FDR) of 5%. The resulting STRING data were then exported into Cytoscape (v3.10.4) [82] to create a protein–protein interaction network, which was then used to identify the top ten hub targets with the CytoHubba plug-in. Furthermore, an express analysis was performed for all the genes encoding proteins predicted as targets for AGEO compounds and pancreatic cancer in Metascape [84] to identify biologically relevant pathways in which these targets could be found.

### 4.13. Molecular Docking Protocol

The crystal structure of the human Hsp90α N-terminal domain (PDB ID: 6LR9) was retrieved from the Protein Data Bank (RCSB PDB) [85]. This structure was selected because it contains a co-crystallised ligand bound within the ATP-binding pocket, enabling direct definition of the docking site and validation of the docking protocol through redocking. As the investigated phytochemicals were expected to interact with the ATP-binding region of Hsp90α, this structure was considered suitable for the present docking study. Prior to docking, the co-crystallised ligand and water molecules were removed, and the receptor was prepared using AutoDock Tools version 1.5.7. Molecular docking simulations were performed using AutoDock Vina implemented in PyRx version 0.8. The docking grid was centred at x = 3.622, y = 34.139, and z = 22.992 Å, with dimensions of 15.539 × 16.834 × 11.920 Å, encompassing the ATP-binding pocket. The docking protocol was validated by redocking the co-crystallised ligand EOR, yielding a root-mean-square deviation (RMSD) of 0.998 Å relative to its crystallographic pose. Docking scores obtained for the investigated phytochemicals were compared with those of the co-crystallised ligands, and for each compound, the docking pose with the lowest predicted binding energy was selected for further analysis. Protein–ligand interactions were analysed and visualised using BIOVIA Discovery Studio Visualizer 2025 (Dassault Systèmes, San Diego, CA, USA).

## 5. Conclusions

The current study reports the chemical and biological assessment of hydroethanolic extracts and EO obtained from *A. graveolens* seeds. The phytochemical composition of the extracts revealed the presence of polar compounds while, conversely, the EO contained mostly lipophilic compounds, particularly, monoterpenes. The in vitro investigation indicated that the hydroalcoholic extracts and AGEO were able to exert dose-dependent cytotoxic effects in PANC-1, A375 and SK-OV cell lines. The antitumour activity reported for A2 and A4 extracts may be associated with their high content of phenolic acids, flavonoids and tannins, while the stronger activity of AGEO was related to its high concentration of lipophilic monoterpenes, mainly carvotanacetone and D-limonene. The reported reduction in cell viability was accompanied by cytoskeletal and nuclear changes as well as reduced OXPHOS efficiency and increased non-phosphorylating respiration, indicating a mitochondrial uncoupling effect. Moreover, the network pharmacology and molecular docking analyses identified HSP90α as a candidate target for the AGEO components, providing a basis for further investigations.

Although the current findings suggest that the observed cytotoxic effects may be caused by alterations of mitochondrial function and apoptosis-related pathways, their direct contribution needs to be established through additional tests such as the determination of specific apoptotic markers. Additionally, the cytotoxic effects of carvotanacetone and D-limonene should be compared with those of AGEO to investigate potential interactions between the AGEO components. Collectively, these findings support the further investigation of *A. graveolens* as a potential source of cytotoxic compounds able to alter cancer bioenergetics and cell survival in vitro.

## Figures and Tables

**Table 1:** Extraction conditions and yield.

Sample Code	Extraction Method	Solvent	Yield	Colour
A1	Maceration in dark (10 days)	40% ethanol	9.39%	brown
A2	Maceration in dark (10 days)	60% ethanol	9.01%	brown
A3	Soxhlet (15 full cycles)	40% ethanol	9.17%	brown
A4	Soxhlet (15 full cycles)	60% ethanol	8.83%	brown
AGEO	Steam hydrodistillation	water	2.80%	pale-yellow

**Table 2:** Polyphenolic profile of *A. graveolens* L. extracts samples A1, A2, A3 and A4 analysed by LC-MS.

Compound Name	Rt (min)	[M-H]^−^ (*m*/*z*)	A1 (µg/mg d.e.)	A2 (µg/mg d.e.)	A3 (µg/mg d.e.)	A4 (µg/mg d.e.)
Caftaric acid	1.96	311	<LOQ	<LOQ	<LOQ	<LOQ
Gentisic acid	2.67	153	ND	ND	ND	ND
Chlorogenic acid	6.45	353	<LOQ	0.63	0.77	1.99
Caffeic acid	6.97	179	<LOQ	<LOQ	0.52	<LOQ
P-coumaric acid	10.56	163	0.61	1.21	0.24	0.29
**Ferulic acid**	**13.91**	**193**	**1.77**	**2.08**	**0.66**	**<LOQ**
Sinapic acid	15.90	223	0.69	<LOQ	3.27	0.49
Hyperoside	21.56	463	ND	ND	ND	ND
Isoquercitrin	22.50	463	ND	ND	ND	ND
Rutin	23.01	609	ND	ND	ND	ND
Rosmarinic acid	24.05	359	ND	ND	ND	ND
Myricetin	24.29	317	ND	ND	ND	ND
**Fisetin**	**25.68**	**285**	**1.31**	**2.11**	**<LOQ**	**0.93**
Quercitrin	26.18	447	ND	ND	ND	ND
Quercetol	30.38	301	ND	ND	ND	ND
Luteolin	32.78	285	ND	ND	ND	<LOQ
Kaempferol	35.63	285	<LOQ	<LOQ	<LOQ	<LOQ
Apigenin	36.91	269	<LOQ	<LOQ	<LOQ	<LOQ

Notes: ND—not detected, below the limit of detection; <LOQ—not quantified, below the limit of quantification.

**Table 3:** Total phenolic content of the *A. graveolens* extracts.

Extract	Corrected Abs	GAE from Curve	TPC
A1	0.3480 ± 0.0010	33.87 ± 0.10 µg/mL	169.4 ± 0.5 mg GAE/g extract
A2	0.3577 ± 0.0006	34.83 ± 0.06 µg/mL	174.1 ± 0.3 mg GAE/g extract
A3	0.3550 ± 0.0010	34.56 ± 0.10 µg/mL	172.8 ± 0.5 mg GAE/g extract
A4	0.3640 ± 0.0010	35.46 ± 0.10 µg/mL	177.3 ± 0.5 mg GAE/g extract

**Table 4:** Total flavonoid content of the *A. graveolens* extracts.

Extract	Corrected Abs	QE from Curve	TFC
A1	0.2017 ± 0.0006	29.57 ± 0.09 µg/mL	5.91 ± 0.02 mg QE/g extract
A2	0.338 ± 0.0010	50.23 ± 0.15 µg/mL	10.05 ± 0.03 mg QE/g extract
A3	0.328 ± 0.0010	48.71 ± 0.15 µg/mL	9.74 ± 0.03 mg QE/g extract
A4	0.4307 ± 0.0015	64.27 ± 0.23 µg/mL	12.85 ± 0.05 mg QE/g extract

**Table 5:** Condensed tannin content of the *A. graveolens* extracts.

Extract	Corrected Abs	CE from Curve	CTC
A1	0.0683 ± 0.0006	26.58 ± 0.21 µg/mL	4.43 ± 0.03 mg CE/g extract
A2	0.0833 ± 0.0006	31.94 ± 0.21 µg/mL	5.32 ± 0.03 mg CE/g extract
A3	0.1027 ± 0.0006	38.85 ± 0.21 µg/mL	6.47 ± 0.03 mg CE/g extract
A4	0.1460 ± 0.0010	54.32 ± 0.36 µg/mL	9.05 ± 0.06 mg CE/g extract

**Table 6:** AGEO chemical composition determined by GC-MS.

Common Name	RI_calc_	RI_lit_	Area_calc_ %
α-Pinene	932	954	0.30
β-Thujene	971	926	0.14
β-Myrcene	987	988	0.33
3-Carene	1008	1009	0.12
**D-Limonene**	**1029**	**1026**	**38.70**
**Carvotanacetone**	**1250**	**1250**	**60.41**

**Table 7:** The calculated IC_50_ values (μg/mL) of *A. graveolens* extracts A1–4 on HaCaT, A375, PANC-1 and SK-OV-3 cells after 24 h treatment.

Cell Line	A1	A2	A3	A4
HaCaT	>1000	>1000	>1000	>1000
A375	>1000	>1000	>1000	>1000
PANC-1	763.1	619.8	>1000	810.2
SK-OV-3	>1000	771.6	>1000	>1000

Values >1000 indicate that the IC_50_ was not reached within the tested concentration range.

**Table 8:** The SI values after the treatment with A1–4.

Cell Line	A1	A2	A3	A4
A375	1.158	1.836	1.314	1.827
PANC-1	3.117	3.138	1.280	2.613
SK-OV-3	1.557	2.521	1.229	1.928

**Table 9:** Characteristics of the top 10 hub nodes in the protein–protein interaction network. Hub targets were ranked using the maximal clique centrality (MCC) algorithm in CytoHubba, while degree, closeness centrality (CC), and betweenness centrality (BC) were calculated using the analyzer tool.

Target	Rank	Degree	CC	BC
STAT3	1	34	0.730	0.295
TNF	2	18	0.540	0.036
MMP9	3	16	0.529	0.014
IL10	4	16	0.519	0.011
PTGS2	5	16	0.519	0.076
PPARG	6	18	0.562	0.041
TLR4	7	18	0.529	0.041
HSP90AA1	8	26	0.628	0.191
MDM2	9	16	0.551	0.045
AR	10	14	0.540	0.022

**Table 10:** Docking scores of the identified phytochemicals and the co-crystallised ligand against Hsp90α (PDB ID: 6LR9).

Compound	Binding Affinity (kcal/mol)
EOR (native ligand)	−8.6
D-limonene	−7.2
Carvotanacetone	−7.2
3-carene	−6
β-thujene	−5.8
β-myrcene	−5.8
α-pinene	−5.4

## Data Availability

The original contributions presented in this study are included in the article. Further inquiries can be directed to the corresponding author.

## Supplementary Materials

The following supporting information can be downloaded at https://www.mdpi.com/article/10.3390/molecules31183337/s1, Table S1. The chemical structures of the compounds identified in the *A. graveolens* L. extract samples; Table S2. The chemical structures of the compounds identified in the *A. graveolens* L. essential oil; Figure S1. TIC of A1–A4 *A. graveolens* L. extract samples analysed by LC-MS; Figure S2. GC-MS profile of dill (*A. graveolens* L.) seed essential oil.

## Abbreviations

The following abbreviations are used in this manuscript:

AGEO	*Anethum graveolens* L. EO
BC	Betweenness centrality
CC	Closeness centrality
CTC	Condensed tannin content
DMEM	Dulbecco’s Modified Eagle Medium
EO	Essential oil
ESI	Electrospray ionization
FDR	False discovery rate
GC-MS	Gas chromatography–mass spectrometry
HPLC	High performance liquid chromatography
LC	Liquid chromatography
MCC	Maximal Clique Centrality
MMP	Mitochondrial membrane potential
MS	Mass spectrometry
OXPHOS	Oxidative phosphorylation
RI_calc_	Retention indices
RI_lit_	Literature retention indices
RMSD	Root-mean-square deviation
SIM	Single ion monitoring
SI	Selectivity index
TFC	Total flavonoid content
TPC	Total phenolic content
