# Supplementary material for: Maceration, Soxhlet Extraction, and Steam Hydrodistillation of Anethum graveolens Seeds: Phytochemical Profiles and Cytotoxic Effects
Source: Molecules. 2026 Sep 20;31(18):3337. doi: 10.3390/molecules31183337 (PMC13609506; doi:10.3390/molecules31183337)
Supplement: Supplementary file 1 [file molecules-31-03337-s001.zip › molecules-4540598-supplementary.pdf]

SUPPLEMENTARY FILE

Maceration, Soxhlet Extraction, and Steam Hydrodistillation of *Anethum graveolens* Seeds: Phytochemical Profiles and Cytotoxic Effects

Christian Goldiș [1], Roxana Racoviceanu [2,3,*], Roxana Negrea-Ghiulai [2,3], Alexandra Mioc [2,3], Alexandra Prodea [2,3], Elisabeta Atyim [2,3], Tamara Maksimovic [2,3], Alexandra T. Lukinich-Gruia [4], Maria-Alexandra Pricop [4,5], Codruța Șoica [2,3]

[1]Faculty of Medicine, "Victor Babeș" University of Medicine and Pharmacy, 2 Eftimie Murgu Square, 300041 Timișoara, Romania
[2]Faculty of Pharmacy, "Victor Babeș" University of Medicine and Pharmacy, 2 Eftimie Murgu Square, 300041 Timișoara, Romania
[3]Research Centre for Experimental Pharmacology and Drug Design (X-Pharm Design), "Victor Babeș" University of Medicine and Pharmacy, 2 Eftimie Murgu Square, 300041 Timișoara, Romania
[4]OncoGen Centre, Clinical County Hospital "Pius Branzeu", Blvd. Liviu Rebreanu 156, 300723, Timisoara, Romania
[5] Department of Applied Chemistry and Environmental Engineering and Inorganic Compounds, Faculty of Industrial Chemistry, Biotechnology and Environmental Engineering, Polytechnic University of Timisoara, Vasile Pârvan 6, 300223, Timisoara, Romania
*Correspondence: babuta.roxana@umft.ro

Table S1. The chemical structures of the compounds identified in the *A. graveolens* L. extract samples

Compound	Chemical structure
Caftaric acid	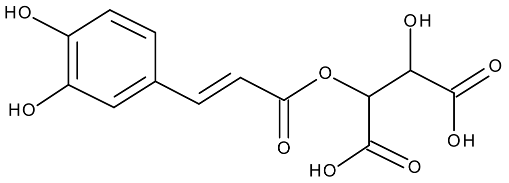
Chlorogenic acid	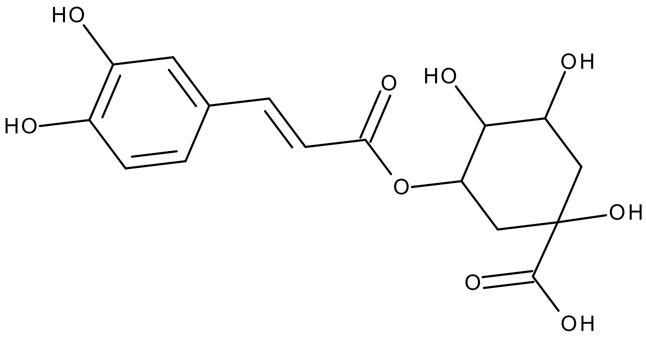

Caffeic acid	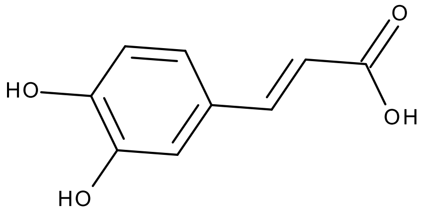
P-coumaric acid	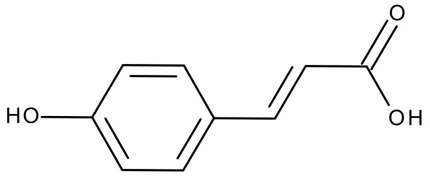
Ferulic acid	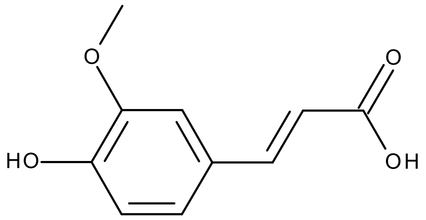
Sinapic acid	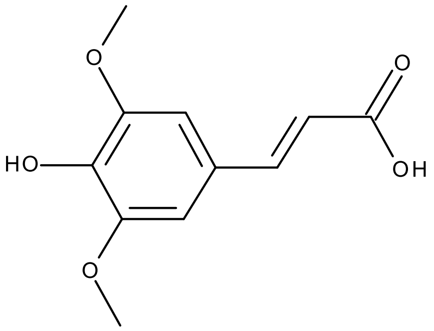
Fisetin	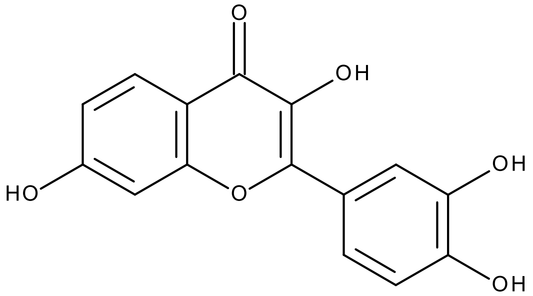
Luteolin	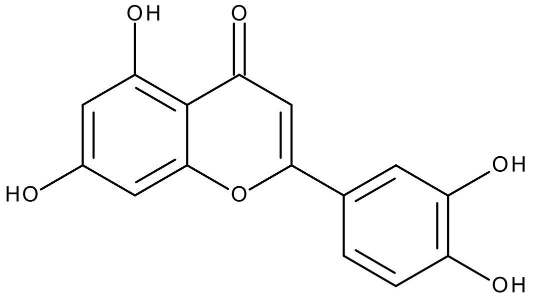

Kaempferol	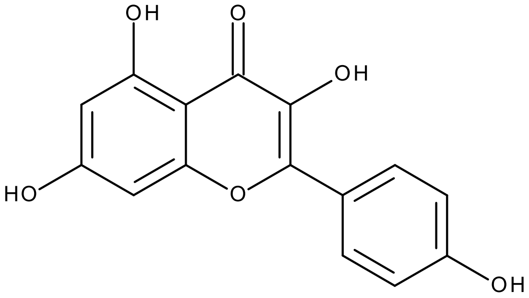
Apigenin	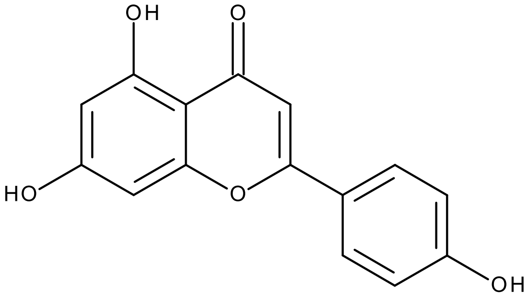

Table S2. The chemical structures of the compounds identified in the *A. graveolens* L. essential oil

**Compound**	**Chemical structure**
α-Pinene	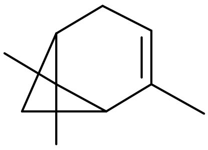
β-Thujene	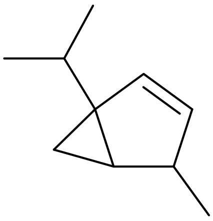
β-Myrcene	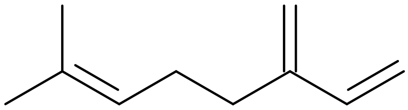
3-Carene	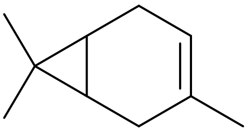
D-Limonene	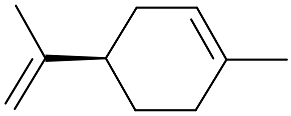

Carvotanacetone	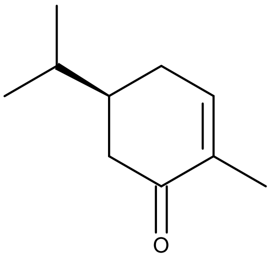

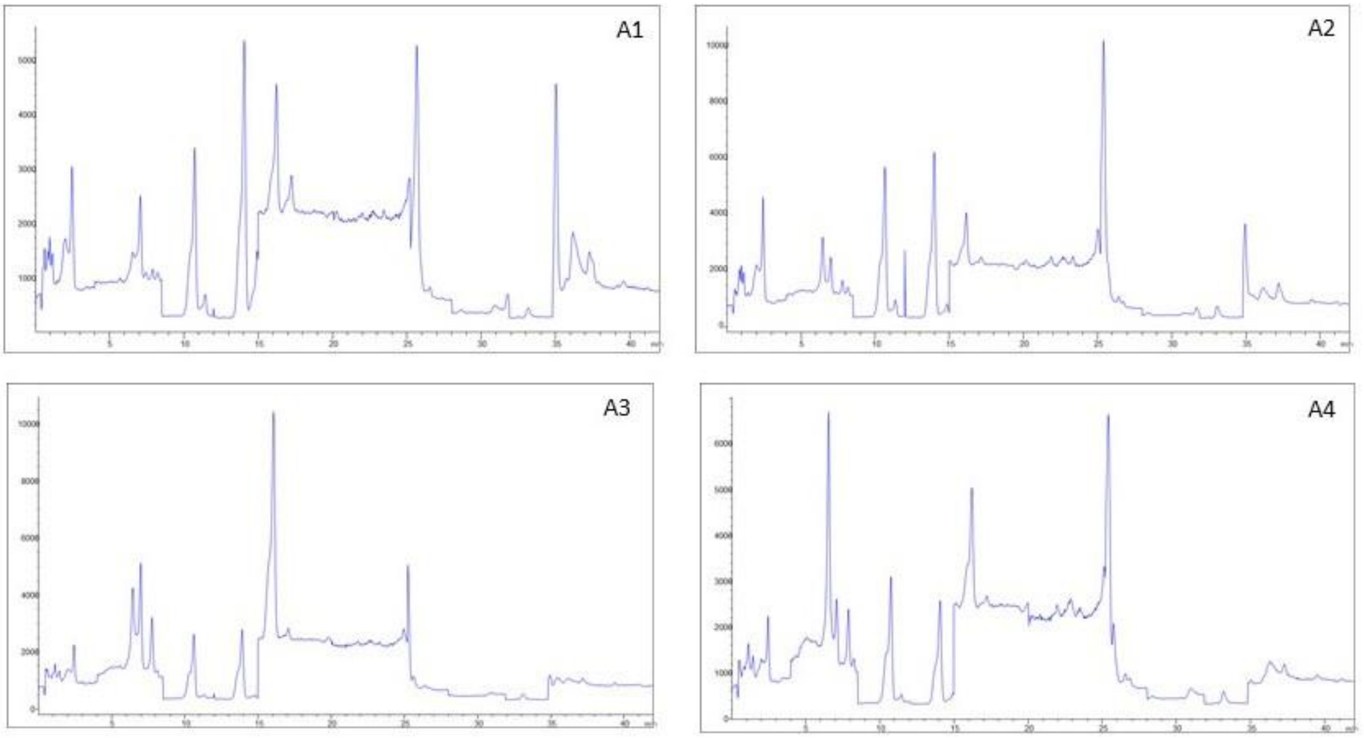

Figure S1.TIC of A1–A4 *A. graveolens* L. extract samples analysed by LC-MS

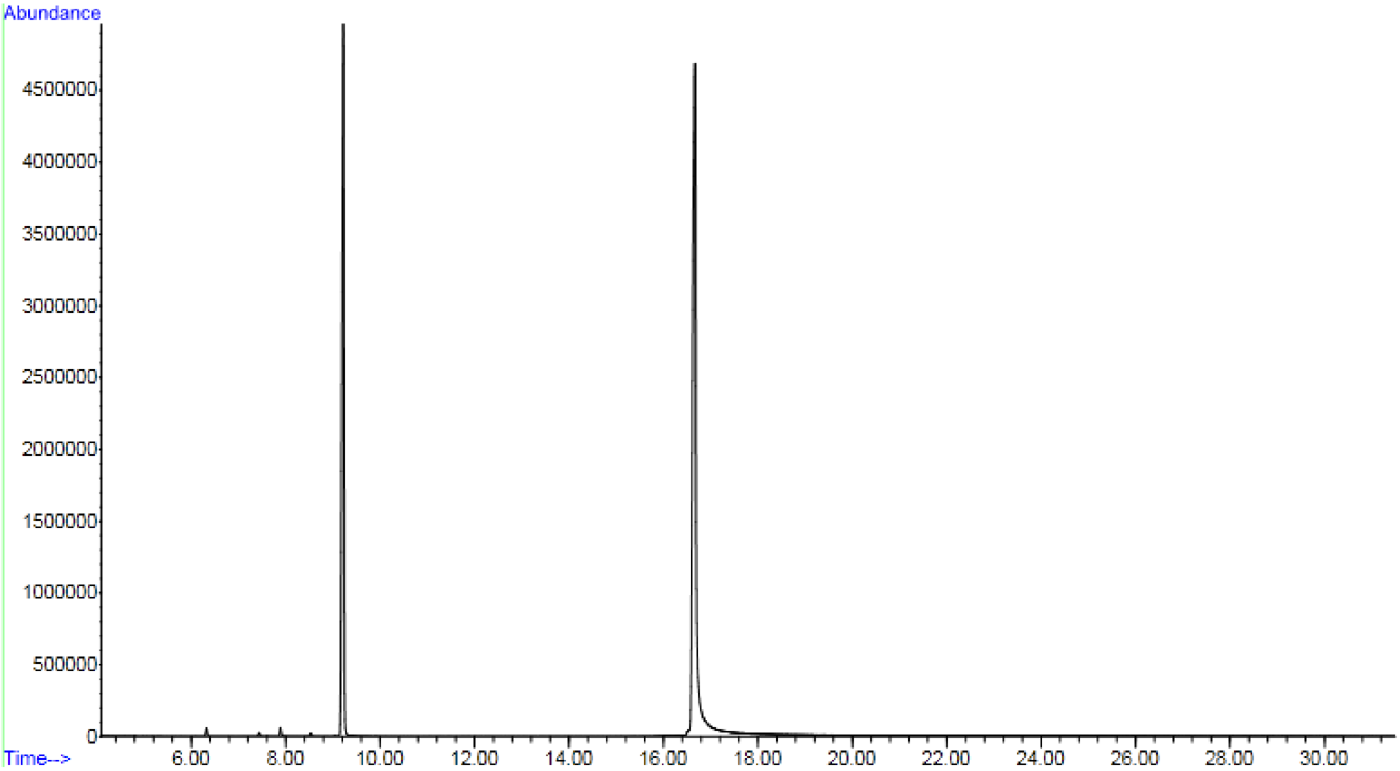

Figure S2. GC-MS profile of dill (*A. graveolens L.*) seed essential oil.
